# Bayesian Nonparametric Models for Multiple Raters: A General Statistical Framework

**DOI:** 10.1017/psy.2025.10035

**Published:** 2025-08-11

**Authors:** Giuseppe Mignemi, Ioanna Manolopoulou

**Affiliations:** 1 Bocconi Institute for Data Science and Analytics, Bocconi University, Milan, Italy; 2 Statistics Department, University College London, Bloomsbury, UK

**Keywords:** Bayesian hierarchical models, Bayesian mixture models, Bayesian nonparametric models, intraclass correlation coefficient, rating models

## Abstract

Rating procedure is crucial in many applied fields (e.g., educational, clinical, emergency). In these contexts, a rater (e.g., teacher, doctor) scores a subject (e.g., student, doctor) on a rating scale. Given raters’ variability, several statistical methods have been proposed for assessing and improving the quality of ratings. The analysis and the estimate of the Intraclass Correlation Coefficient (ICC) are major concerns in such cases. As evidenced by the literature, ICC might differ across different subgroups of raters and might be affected by contextual factors and subject heterogeneity. Model estimation in the presence of heterogeneity has been one of the recent challenges in this research line. Consequently, several methods have been proposed to address this issue under a parametric multilevel modelling framework, in which strong distributional assumptions are made. We propose a more flexible model under the Bayesian nonparametric (BNP) framework, in which most of those assumptions are relaxed. By eliciting hierarchical discrete nonparametric priors, the model accommodates clusters among raters and subjects, naturally accounts for heterogeneity, and improves estimates’ accuracy. We propose a general BNP heteroscedastic framework to analyze continuous and coarse rating data and possible latent differences among subjects and raters. The estimated densities are used to make inferences about the rating process and the quality of the ratings. By exploiting a stick-breaking representation of the discrete nonparametric priors, a general class of ICC indices might be derived for these models. Our method allows us to independently identify latent similarities between subjects and raters and can be applied in *precise education* to improve personalized teaching programs or interventions. Theoretical results about the ICC are provided together with computational strategies. Simulations and a real-world application are presented, and possible future directions are discussed.

## 1 Introduction

Rating procedure is crucial in several applied scientific fields, such as educational assessment (Childs & Wooten, 2023; Chin et al., 2020), psychological and medical diagnoses (D’lima et al., 2024; Królikowska et al., 2023; Li et al., 2022), emergency rescue (Albrecht et al., 2024; Lo et al., 2021) or grant review process (Cao et al., 2010; Sattler et al., 2015). It implies that an observer, commonly called a rater (e.g., teacher, doctor), assesses some subject attribute or latent ability (e.g., student proficiency, patient severity) on a rating scale. Raters’ variability might pose reliability concerns and uncertainty about the quality of ratings (Bartoš & Martinková, 2024; Mignemi et al., 2024; Ten Hove et al., 2021). Several statistical methods have been proposed to address these issues, they aim to assess or improve the accuracy of ratings (Casabianca et al., 2015; Gwet, 2008; Martinková et al., 2023; McGraw & Wong, 1996; Nelson & Edwards, 2015). Multilevel modelling serves as a natural statistical framework for rating data since subjects are either nested within raters or crossed with them (Ten Hove et al., 2021). These models (e.g., one-way or two-way ANOVA, hierarchical linear or generalized linear models) decompose the total variance of observed ratings according to different sources of variability, i.e., subjects and raters (see Martinková & Hladká, 2023, chapter 4, for an overview). The observed rating is commonly broken down into different effects, for instance, the effect of the subject (i.e., true score, latent ability; Lord & Novick, 1968), the effect of the rater (i.e., rater’s systematic bias) and a residual part (McGraw & Wong, 1996; Shrout & Fleiss, 1979). This allows us to jointly estimate the subject true score and the reliability of ratings, which is generally referred to as the proportion of total variance due to the subjects’ variability (McGraw & Wong, 1996; Werts et al., 1974).

Several methods have been proposed to analyze rating data under the Item Response Theory (IRT) framework, such as the Generalized Many Facet Rasch Models (GMFRMs; Linacre, 1989; Uto et al., 2024; Uto & Ueno, 2020), the Hierarchical Raters Models (HRMs; DeCarlo et al., 2011; Molenaar et al., 2021; Nieto & Casabianca, 2019; Patz et al., 2002) or the Generalized Hierarchical Raters Models (GHRMs; Muckle & Karabatsos, 2009). These models jointly estimate the subject’s latent ability, rater effects (e.g., systematic bias and reliability), and item features (i.e., difficulty, discrimination). They typically rely on the assumption that subjects’ latent abilities are independent and identically distributed (i.i.d.) from a normal distribution. Other recent research lines concentrate on modeling and estimation issues in the presence of subjects’ and raters’ heterogeneity (Martinková et al., 2023; Mutz et al., 2012; Sattler et al., 2015; Ten Hove et al., 2022). These works model systematic differences among subjects or raters are to allow more accurate estimates and detailed information about the rating procedure. Individual subjects’ or raters’ characteristics may affect rating reliability, so that more flexible models result in separate reliability estimates (Martinková et al., 2023). Recent models have been proposed to address this issue under a parametric multilevel modeling framework (Erosheva et al., 2021; Martinková et al., 2023; Martinkova et al., 2018; Mutz et al., 2012) in which heterogeneity is addressed as a covariate-dependent difference among subjects and subject- and rater-specific effects are assumed to be i.i.d from a normal distribution. The normality assumption made under all the aforementioned models might be unrealistic under a highly heterogeneous scenario in which possible clusters among subjects or raters might be reasonably expected and the conditional density of the respective effects might be multimodal (Paganin et al., 2023; Verbeke & Lesaffre, 1996; Yang & Dunson, 2010). Such patterns have emerged from real data, showing that both the conditional densities of subjects’ latent ability (e.g., Uto et al., 2024) and raters’ systematic bias (e.g., Muckle & Karabatsos, 2009) might be multimodal and the normality assumption violated. In these cases, the data exhibit two levels of heterogeneity. The first, known as *individual* heterogeneity, captures the differences between individuals; the second, referred to as *population* heterogeneity, pertains to the differences between clusters. Although parametric mixture models might represent a suitable solution, the number of mixture components needs to be fixed. Models with different numbers of components have to be fitted and model selection techniques are required to identify the optimal number of clusters (Bartholomew et al., 2011).

### 1.1 Our contributions

Our proposal aims to overcome these restrictions under a Bayesian nonparametric (BNP) model, which naturally accommodates subgroups among students and raters and allows less restrictive distributional assumptions on the respective effects (Ferguson, 1973; Ghosal & van der Vaart, 2017; Hjort et al., 2010). BNP inference has led to new developments and advances during the last decades in psychometrics (Cremaschi et al., 2021; Karabatsos & Walker, 2009; Paganin et al., 2023; Roy et al., 2024; San Martín et al., 2011; Tang et al., 2017; Wang & Kingston, 2020; Yang & Dunson, 2010), but few contributions have been proposed for the analysis of rating data (DeYoreo & Kottas, 2018; Kottas et al., 2005; Mignemi et al., 2024; Savitsky & Dalal, 2014). We provide a flexible statistical framework for rating models in which latent heterogeneity among subjects and raters is captured with the stochastic clustering induced by the Dirichlet Process Mixture (DPM) placed over their respective effects. Modelling subjects’ and raters’ effect parameters as an infinite mixture of some distribution family (e.g., Normal, Gamma) enables the model to account for possible multimodality without specifying the number of mixture components (De Iorio et al., 2023; Yang & Dunson, 2010). Although previous works have raised questions about the identifiability of the parameters in BNP IRT models San Martín et al. (2011), theoretical results by (Pan et al., 2024) have recently shown that BNP IRT models (e.g., 1PL) are identifiable.

Under the general case of a two-way design (McGraw & Wong, 1996), we specify a measurement model for the subject latent ability (e.g., student proficiency) in which the rater’s systematic bias (i.e., severity) and reliability are consistently estimated. This makes our method more relevant for subject scoring purposes than the other BNP models proposed for the analysis of rating data (DeYoreo & Kottas, 2018; Kottas et al., 2005; Savitsky & Dalal, 2014). Our proposal may be suitable both for balanced (i.e., when all raters score each subject; Nelson & Edwards, 2010, 2015) and unbalanced designs (i.e., when a subset of raters scores each subject; Martinková et al., 2023; Ten Hove et al., 2022). Furthermore, we propose a Semiparametric model as a nested version of the BNP in which raters’ effects are i.i.d. from a unimodal distribution. Very small rater sample sizes may not reasonably be considered representative of the overall rater population, making the semiparametric specification a potentially more suitable choice.

The advantages of the proposed method are manyfold. First, it relies on more relaxed distributional assumptions for the subjects’ and raters’ effects, allowing for density estimation using mixtures (Escobar & West, 1994; Ghosal et al., 1999) and preventing model misspecification issues (Antonelli et al., 2016; Walker & Gutiérrez-Peña, 2007). As recently argued by Tang et al. (2017), BNP priors might be helpful in assessing the appropriateness of common parametric assumptions for psychometrics models and represent a solution under their violation (Antoniak, 1974; Ferguson, 1973). Second, it naturally enables independent clustering of subjects and raters, bringing more detailed information about their latent differences (De Iorio et al., 2023; Mignemi et al., 2024). This allows the joint analysis of *individual* and *population* heterogeneity of both subjects and raters. This aspect might be beneficial in the context of *precise education* (Coates, 2025; Cook et al., 2018), where information about individual and cluster differences might be used for implementing more personalized educational programs or interventions (Hart, 2016; Henderson et al., 2020). Third, exploiting a stick-breaking representation of the Dirichlet Process(DP) (Ghosal & van der Vaart, 2017; Ishwaran & James, 2001), a general class of ICC indices might be derived, and different indices might be computed according to distinct clusters of subjects or raters. Fourth, it is readily extended to account for coarse or ordinal ratings (Goel & Thakor, 2015; Lockwood et al., 2018). Fifth, the general hierarchical formulation of our model allows comparisons with other methods and further extensions under unifying modelling frameworks (e.g., generalized linear latent and mixed model, GLLAMM Rabe-Hesketh & Skrondal, 2016). This facilitates a straightforward communication between different statistical fields and a wider application of the BNP method.

Model parameters are learned through full posterior sampling. Since most of the parameters in the model have conjugate prior distributions, full conditional Gibbs sampling is possible for most of the parameters (Ishwaran & James, 2001). Nonetheless, few parameters do not have conjugate priors and a derivatives matching technique is involved to approximate the full conditional (Miller, 2019).

### 1.2 Outline of the article

The outline of the article is as follows: we present the general framework and introduce the model in Sections 2.1–2.3, respectively; different approximate ICC indices are derived in Section 3 and a reduced model for one-way designs is detailed in Section 4; prior elicitation and posterior sampling are discussed and presented in Section 5; simulations and real-world applications are illustrated, respectively, in Section 6 and Section 7; the model extension for coarse ratings and is presented in Section 8, along with some numerical results from real and generated data. Advantages and limitations of the proposal are discussed in Section 9. Further BNP extensions, proofs for ICCs indices, and additional plots are given in the Appendices. Additional results on balanced design in small sample sizes, technical details on out-of-sample predictive performance assessment and posterior computation for this class of models are presented in the Supplementary Material. We provide an R package RatersBNP to facilitate direct usage by researchers and practitioners of our method. Code and Supplementary Material are available online through the link: https://osf.io/3yx4j/?view_only=98c600198a6b4807878989765118f97e.

## 2 BNP rating model

### 2.1 General framework

Several model specifications have been proposed for different data structures and designs(Gwet, 2008; Shrout & Fleiss, 1979; Ten Hove et al., 2022). One-way designs are preferred when rater differences are typically considered as noise (Martinková et al., 2023), whereas two-way designs are usually involved if the rater’s effect needs to be identified (Casabianca et al., 2015; Mignemi et al., 2024). Balanced designs require each subject to be rated by all the raters, while in an unbalanced design each subject is only rated by a generally small subset of them (Ten Hove et al., 2021). Raters might be considered either fixed or random (i.e., drawn from the population) depending on the inference the researcher might be interested in (Koo & Li, 2016).

The unbalanced two-way design with random raters is considered a general case to present our model. The reasons for this choice are both theoretical and practical. We aim to provide a comprehensive statistical framework for modeling the dependency of ratings on different categorical predictors (i.e., subjects’ and raters’ identities). This setting is a neat compromise between the one-way design, which implies only one categorical predictor (i.e., subject identity), and more complex dependency structures that involve more than two identities (i.e., several categorical predictors). However, an example of these extensions is given in Appendix A.2. Indeed, our proposal might be alternatively reduced or extended to be suitable for these different levels of complexity. The unbalanced design implies some sparsity in the co-occurrence between subjects and raters and each subject is rated only by a small subset of raters (Papaspiliopoulos et al., 2019; Papaspiliopoulos et al., 2023), as a consequence each rater might score a different number of subjects. This makes the framework general and flexible, it might be seen as an extension of cross-classified models in which uncertainty is modeled also hierarchically. From a practical perspective, our choice is reasonable since many large studies and applications use unbalanced designs to distribute the workload across different raters (Ten Hove et al., 2022).

### 2.2 Preliminaries on Bayesian nonparametric inference

In this subsection, we briefly review some basic preliminaries on BNP inference providing here a very general framework which is detailed in Sections below (refer to Ghosal & van der Vaart, 2017 and Hjort et al., 2010 for exhaustive treatments).

Suppose 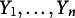 , are observations (e.g., ratings), with each 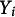 taking values in a complete and separable metric space 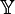 . Let 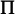 denote a prior probability distribution on the set of all probability measures 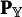 such that (1)

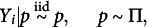

for 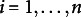 . Here *p* is a random probability measure on 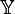 and 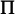 is its probability distribution and might be interpreted as the prior distribution for Bayesian inference (De Blasi et al., 2015). The inferential problem is called parametric when 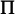 degenerates on a finite-dimensional subspace of 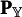 , and nonparametric when the support of 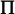 is infinite-dimensional (Hjort et al., 2010, chapter 3). To the best of our knowledge, the vast majority of the contributions present in rating models literature (Bartoš & Martinková, 2024; Casabianca et al., 2015; Martinková et al., 2023; Martinkova et al., 2018; Nelson & Edwards, 2010, 2015; Ten Hove et al., 2021, 2022; Zupanc & Štrumbelj, 2018) are developed within a parametric framework making use of a prior that assigns probability one to a small subset of 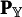 . Although Mignemi et al. (2024) recently proposed a Bayesian semi-parametric (BSP) model for analyzing rating data. Even if they relax the normality assumption for the rater effect (i.e., the systematic bias), normality is still assumed for the subject true score distribution. This strong prior assumption is overcome through a BNP approach (Ghosal & van der Vaart, 2017) in the present work.

*Dirichlet processes.* For the present proposal, we assume 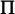 to be a discrete nonparametric prior and correspond to a DP which has been widely used in BNP psychometric research (Cremaschi et al., 2021; Karabatsos & Walker, 2009; Paganin et al., 2023; Yang & Dunson, 2010). Given 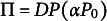 , *p* is a random measure on 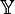 following a DP with concentration parameter 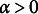 and base measure 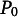 . This implies that for every finite measurable partition 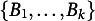 of 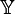 , the joint distribution 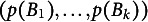 follows a *k*-variate Dirichlet distribution with parameters 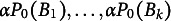 : (2)

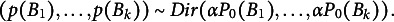

The base measure 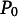 is our *prior guess* at *p* as it is the prior expectation of the DP, i.e., 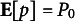 . The parameter 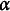 (also termed precision parameter) controls the concentration of the prior for *p* about 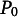 . In the limit of 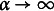 , the probability mass is spread out and *p* gets closer to 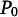 ; on the contrary, as 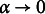 , *p* is less close to 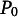 and concentrates at a point mass.

*Dirichlet process mixtures.* Given the discrete nature of the DP, whenever 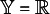 it is not a reasonable prior for the real-valued random variable *Y*. Nonetheless, it might be involved in density estimation through hierarchical mixture modeling (Ghosal & van der Vaart, 2017). Let 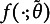 denote a probability density function for 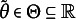 , we modify (1) such that for 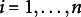 : (3)

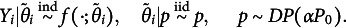

The realizations of the DP are almost surely (a.s.) discrete which implies a positive probability that 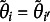 , for 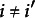 . Indeed, a random sample 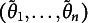 from *p* features 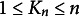 different unique values 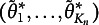 and leads to a random partition of 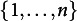 into 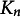 blocks such that 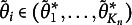 for 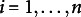 . This naturally induces a mixture distribution for the observations 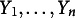 with probability density: (4)

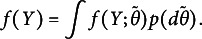

To provide some intuition, by using a DP as a prior for an unknown mixture distribution we mix parametric families nonparametrically (Gelman et al., 2014). This model specification introduced by Lo (1984) and termed DPM provides a BNP framework to model rating data.

### 2.3 Proposed model

Consider a subject 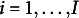 , whose attribute is independently scored by a finite random subset of raters 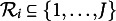 on a continuous rating scale. We assume that the observed rating 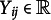 depends independently on subject *i* and rater 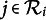 . The effect of the former is interpreted as *i*’s true score and is the rating procedure’s focus. We let the residual part, that is the difference between the true and the observed score, depend on rater *j*’s effects, i.e., systematic bias and reliability.

*Modelling rating* 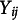 . We specify the following decomposition of rating 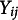 : (5)

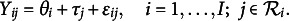

Here 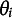 captures the subject *i*’s latent “true” score and 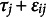 is the difference between the observed and the true score, representing the error of rater *j*. The true score 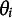 might be regarded as the latent consensus across the raters 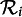 on the attribute of subject *i*.We assume these terms to be mutually independent.

*Modelling subject’s true score.* For each subject 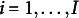 we assume that the true score 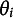 is independently distributed following a normal distribution with mean 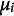 and variance 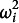 : (6)

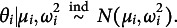

Here 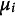 is the mean of subject *i*’s true score, 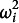 is its variability and we assume them to be independent. Conditional on the rater’s error, higher values of 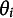 imply higher levels of the subjects’ attribute (e.g., higher student proficiency); on the contrary lower values indicate poor levels of their attribute (e.g., poor student proficiency).

We specify a DP prior with precision parameter 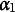 and base measure 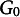 for the pair 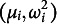 , 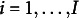 : (7)

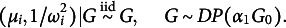

**Figure 1:**
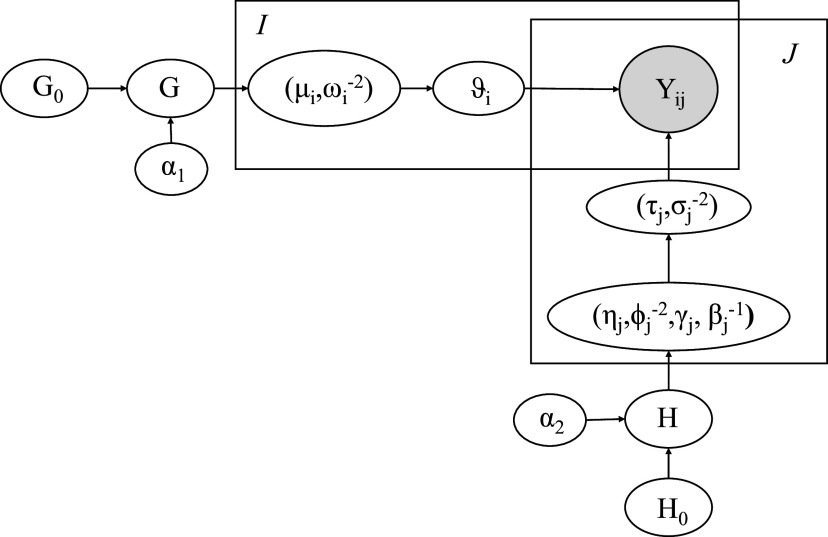
Graphical representation of the dependencies implied by the model. The boxes indicate replicates, the four outer plates represent, respectively, subjects and raters, and the inner grey plate indicates the observed rating.

We choose 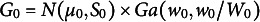 , where 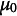 and 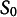 are the mean and variance of the normal distribution and 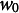 and 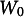 are, respectively, the shape and the mean parameters of the gamma. We note that *G* is a.s. discrete with a non-zero probability of ties, such that different subjects will share the same values of 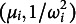 with a probability greater than zero, that is 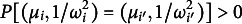 , for 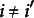 . This discreteness property naturally induces clustering across subjects and leads to a location-scale DPM prior for 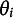 . That is, this formulation can capture clusters of subject abilities. Figure 1 shows the hierarchical dependence of subjects’ true scores.

*Modelling rater’s bias and reliability.* For each rater 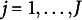 , who scores a subset of subjects 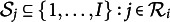 , the difference between the observed rating 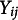 and the subject’s true score 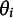 , 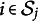 , is decomposed into the rater effects 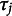 and 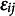 (5), assuming 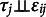 . We model 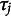 to be normally distributed with mean 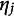 and variance 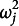 : (8)

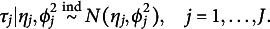

Here 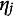 and 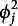 are the mean and the variance of the rater *j*’s effect 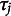 . It captures *j*’s specific systematic bias, i.e., the mean difference between the observed rating 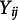 and the subject’s true score 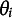 , 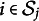 . Given two raters such that 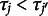 , *j* is said to be more strict and expected to give systematically smaller ratings than *j* on average.

The residual term 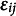 is assumed to be i.i.d. for 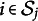 following a normal distribution with zero mean and variance 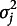 . We let this parameter vary across raters and assume 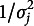 follows a gamma distribution with shape and rate parameters 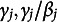 , respectively: (9)

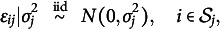


(10)

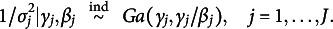

Under this parametrization, 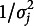 is the rater *j*’s specific reliability with mean 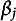 and 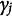 is the shape parameter. We prefer this parametrization for interpretability purposes, which implies a simpler notation below. Conditional on subjects’ true score 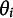 , 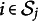 , larger values of 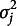 imply more variability across the ratings given by *j* and might be interpreted as a poorly consistent rating behaviour. On the contrary, smaller values of 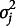 indicate less variability and higher consistency for *j* across subjects. As a consequence, the parameter 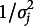 might be equivalently referred to as the rater-specific precision.

We specify a DP prior with concentration parameter 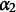 and base measure 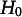 for the four-dimensional vector 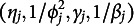 , 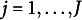 : (11)

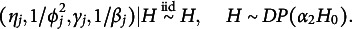

We assume mutual independence for the elements of the vector and choose 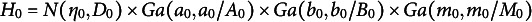 , where 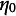 and 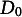 are mean and scale parameters, respectively; 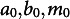 are shape parameters and 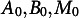 are mean parameters. This formulation induces a DPM prior for raters’ bias and reliability 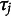 and 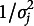 . Figure 1 gives a graphical representation of the model. The independence assumption might be relaxed by employing a suitable multivariate base measure accounting for possible dependencies among the four elements of the vector. However, this implies a more complex specification, which is beyond the purpose of this work. Further constraints on raters’ systematic bias 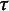 are needed for identifiability purposes which are discussed in Section 2.5, after presenting the stick-breaking representation.

### 2.4 Stick-breaking representation

The random probability measures *G* and *H* are assigned discrete priors, as a consequence they might be represented as a weighted sum of point masses: (12)

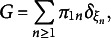


(13)

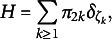

where the weights 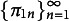 and 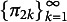 take values on the infinite probability simplex and 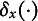 stands for the Dirac measure and denotes a point mass at *x*. Note that, we index the components of the infinite mixture (12) corresponding to the subjects with 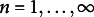 , whereas 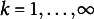 is used for that corresponding to the raters (13). The random vectors 
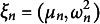
 , 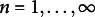 are i.i.d. from the base measure 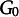 , 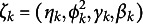 , 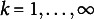 are i.i.d. from the base measure 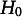 , and both vectors are assumed to be independent of the corresponding weights. This makes clear why the expectations of the 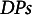 are 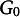 and 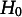 , respectively, and are said to be our *prior guess* at *G* and *H* (see Section 2.2).

This discreteness property of the 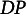 allows us to define *G* and *H* through the stick-breaking representation introduced by Sethuraman (1994): (14)

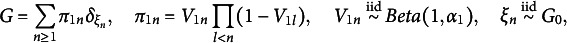

and (15)

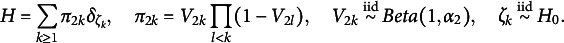

This construction of the 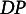 implies that, for each subject 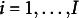 , 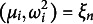 with probability 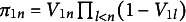 . Equivalently, for each rater 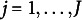 , the probability that 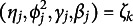 is given by 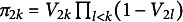 .

*Moments of student latent true score* 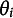 . The mean and the variance of the subject’s true score 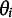 , 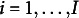 , under a 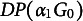 prior are: (16)

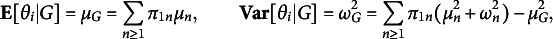

where 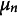 and 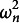 are the mean and the variance of 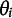 for the *n*th component of the mixture. Here 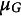 is the weighted average across components and captures the mean true score across subjects. The parameter 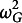 is the conditional variance of the infinite mixture and indicates the variability of true scores across subjects.

*Moments of raters’ bias* 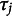 . The mean and the variance of the rater’s bias 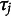 , 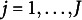 , under a 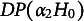 prior are: (17)

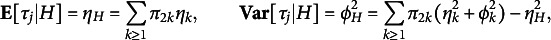

where 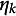 and 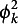 are the mean and the variance of 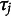 for the *k*th component of the mixture. Here 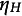 and 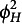 capture the mean and the variance of the systematic bias within the general population of raters.

*Moments of raters’ reliability* 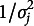 . Raters’ residual mean is fixed to zero by the model (9), that is 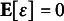 ; mean and variance of raters reliability 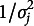 under a 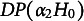 prior are: (18)

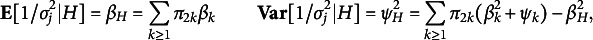

where 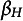 captures raters’ weighted average reliability and 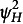 indicates the total reliability variance across them. Here 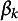 and 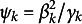 are, respectively, the mean and the variance of 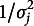 for the *k*th component of the mixture.

Note that we model the independent rater’s features, i.e., bias and reliability, by placing the same 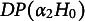 prior. In other terms, 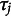 and 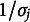 are two independent elements of the same vector drawn from *H*.

*Finite stick-breaking approximation.* The recursive generation defined in (14) and (15) implies a decreasing stochastic order of the weights 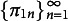 and 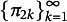 as the indices *n* and *k* grow. Considering the expectations 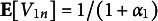 and 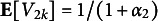 it is clear that the rates of decreasing depend on the concentration parameters 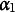 and 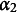 , respectively. Values of these parameters close to zero imply a mass concentration on the first couple of atoms, with the remaining atoms being assigned small probabilities; which is consistent with the general formulation of the 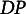 discussed in Section 2.2. Given this property of the weights, in practical applications the infinite sequences (12) and (13), are truncated at enough large values of 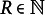 : (19)

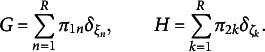

We use this finite stick-breaking approximation proposed by Ishwaran & James (2001) to let 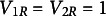 , and discard the terms 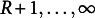 , for *G* and *H*.

The moment formulas (16), (17), and (18) are readily modified accordingly to the truncation and computed as finite mixture moments.

*Nested versions.* Semiparametric nested versions of the BNP model might be specified in which alternatively *G* or *H* are degenerate on a single component and 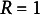 for one of them in the finite approximation. That is, subjects or raters are all clustered together. For instance, for very small values of *J* (i.e., raters’ sample size), raters might not be reasonably considered a representative sample of their population and limited information is available for drawing inference about it. Under these scenarios, raters’ effects might be assumed to be i.i.d. from a normal distribution.

### 2.5 Semi-centered DPM

Hierarchical models (e.g., GLMM, Linear Latent Factor models), might suffer from identifiability issues, and constraints on the latent variable distributions are needed for consistently identify and interpret model parameters (Bartholomew et al., 2011; Gelman & Hill, 2006; Yang & Dunson, 2010). More specifically, under the linear random effects models a standard procedure to achieve model identifiability is to constrain the mean of the random effects to be zero (Agresti, 2015). We aim to consistently involve the same mean constraint for our proposal and allow straightforward and interpretable comparisons between the parametric and the nonparametric models. Similar to Yang & Dunson (2010), we encompass a DPM-centered prior such that the expected value of the rater systematic bias is fixed to zero, 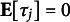 , for 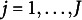 .

Since the rating process focuses on the subjects’ true scores, it might be more reasonable to centre the DPM for the raters’ effects and let the model estimate the mean of the true scores 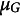 . Given that the mean of the raters’ residual is fixed to zero in (9), the mean raters’ bias needs to be fixed. We adapt the centering procedure based on a parameter-expanded approach proposed by Yang et al., 2010 and Yang & Dunson, 2010 to our proposal. We specify a semi-centered DPM (SC-DPM) involving an expansion in raters’ systematic bias 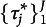 , such that their mean 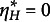 a.s. The expanded-parameters (8) can be expressed as (20)

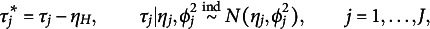

and the decomposition of rating 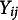 (5) becomes (21)

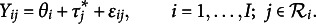

Given the location transformation in (20) the expectation of the expanded parameters is zero: (22)

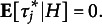

It is worth noting that the centering needs only to concern the location of the systematic bias and not its scale as it is in the centered-DPM introduced by Yang & Dunson (2010), which explains the term “semi-centring” adopted here to avoid confusion. Accordingly, under the semiparametric specifications, the only location of the parametric distribution needs to be fixed; a zero mean normal distribution might be a suitable solution.

## 3 BNP intra-class correlation coefficient

Intra-class correlation coefficient (ICC) is widely used in applied statistics to quantify the degree of association between nested observations (Agresti, 2015; Gelman et al., 2014) and to get relevant information about the level of heterogeneity across different groups (Mulder & Fox, 2019). Indeed, it is commonly applied in psychometrics to assess the consistency of ratings given by different raters to the same subject (Erosheva et al., 2021; Martinková et al., 2023; Nelson & Edwards, 2010, 2015; Ten Hove et al., 2021, 2022). We provide a within-subject correlation structure (for any subject and a given raters pair) 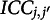 based on the BNP model presented in Section 2.3. This formulation relates to those proposed in psychometric literature regarding the 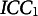 (e.g., Bradlow et al., 1999; De Boeck, 2008; Erosheva et al., 2021; Fox & Glas, 2001; Shrout & Fleiss, 1979; Werts et al., 1974), but doesn’t rely on strong distributional assumptions and naturally accommodates for both subjects and raters sub-populations. We also propose a lower bound 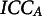 for the expected ICC which might be used for inference purposes about the general population of raters. An exact formula for the ICC suitable for the reduced one-way designs is proposed in Section 4.1. We propose a BNP class of ICCs which are a generalization of those recently proposed by (Lin et al., 2025) and Ten Hove et al. (2021) under the generalizability theory.

The paragraphs below provide preliminary information on computing the ICC under a parametric framework necessary to detail the BNP extension.

*Parametric ICC.* Under a parametric standard framework, i.e., equipping the parameters with finite-dimensional priors, the ICC is defined as the proportion of variance of the ratings due to the subjects’ true score: (23)

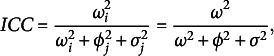

assuming 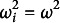 , for 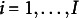 ; 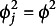 and 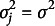 , for 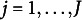 . Given two raters 
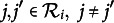
 who rate the same subject *i*, the ICC is the correlation between the ratings 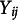 and 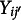 . Note that under this formulation 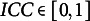 , it can not capture any negative correlations. This index is also interpreted as the inter-rater reliability of a single rating and is also indicated by 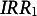 (see Erosheva et al., 2021 for further details). The homoscedastic assumption may be relaxed and raters’ residual variance might be let to vary across raters according to (9) and (10), given 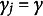 and 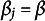 for 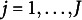 .

Given that 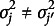 for 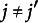 , it is possible to compute as many ICCs indices as possible pairs of raters, i.e., 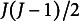 . In such cases the resulting 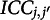 is the conditional correlation between the the ratings given to a random subject by raters *j* and 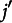 , given the other parameters: (24)

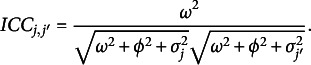

A more general index accounting for all raters’ residual variance might be more useful in applications. Despite the expected ICC, i.e., 
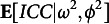
 , might represent a neat solution, it is not available in a close form and the posterior mean taken over the MCMC might be prohibitive in large scale assessments since there are 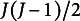 ICCs indices to compute for each iteration. An alternative index that might be readily computed is the ICC between two raters with average reliability. That is, we replace 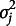 with its expectation, i.e., 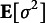 : (25)

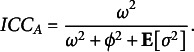

It gives the correlation between the ratings given to the same random subject 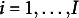 by two random raters 
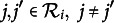
 , satisfying 
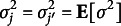
 . That is the correlation between two ratings given to the same random student by two raters having an average reliability level. We note that they are different quantities: the expected pairwise ICC and the pairwise ICC between two mean reliable raters. Nonetheless, relying on a theoretical result that is given below, we can use the 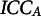 to have information about the other.

Given that the rater’s reliability is assumed to follow a gamma distribution (9), the inverse follows an inverse gamma distribution 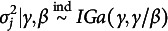 for 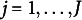 , whose expected value is only defined for 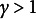 . In such cases we reparametrize (9): (26)

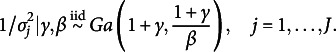

This specification ensures the expectation of raters’ residual variance to be defined for any 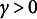 and implies (27)

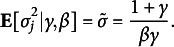

It is the mean raters’ residual variance and its derivation is given in the Supplementary Material. The 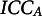 under the new parametrization is (28)

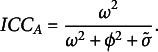

**Figure 2:**
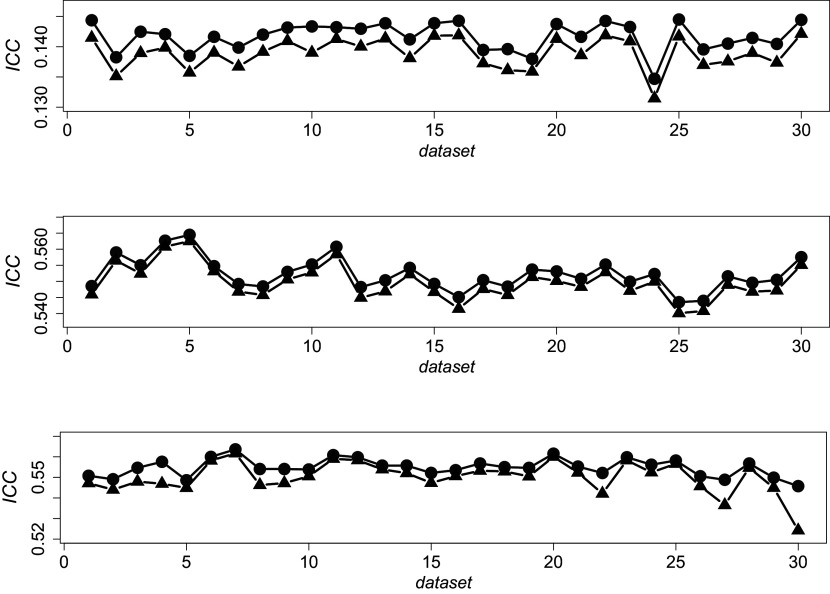
Illustrative examples of empirical 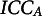 and 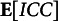 across independent datasets and under different reliability scenarios. The grey balls indicate the mean pairwise 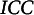 between each rater and the others; the black triangles represent the computed 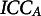 .

Figure 2 shows the difference between the empirical mean pairwise 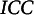 between each rater (red solid line) and the others and the computed 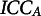 (blue solid line) across independent datasets and different reliability scenarios. The mean difference between these two indices is consistently tight, and it seems to be narrower at increasing reliability levels.

*BNP ICC.* The moments defined in (16), (17), and (18) account for heterogeneous populations of subjects and raters and can be used to compute a flexible ICC.

Proposition 1. Given a random subject 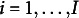 , independently scored by two random raters 
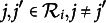
 , the conditional correlation between the scores 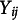 and 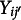 is (29)

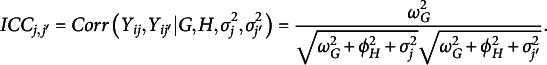

The proof is reported in Appendix B. However, a more general index, unconditioned on specific raters’ parameters, might be more useful in practice. For this reason, we propose a 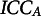 index for this BNP class of models. To this aim, the variance of subjects’ true score 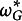 and the variance of raters’ systematic bias 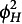 can be directly plugged into the ICC formula. Since we have heteroscedasticity across raters, we need to take the expectation of raters’ residual variance 
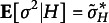
 . Similarly to the above parametric case, we reparametrize (10) with: (30)

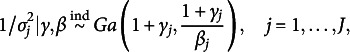

and define: (31)

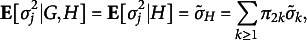

where 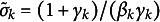 is the mean residual variance for the *k*th component of the infinite mixture. As a result, the 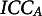 for the BNP models might be computed as reported below.

Proposition 2. Given a random subject 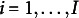 , independently scored by two random raters 
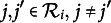
 , satisfying 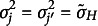 :

- the conditional correlation between the ratings 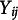 and 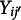 is (32)

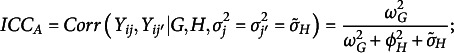

- the 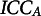 is the lower bound of the conditional expectation of the correlation between the ratings 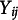 and 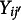 (ICC): (33)

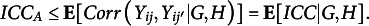

The proofs are reported in Appendix B. The index therefore accounts for the heterogeneity of the two populations (subjects and raters). It reduces to the parametric 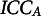 (23) whenever 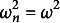 , for 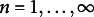 ; 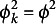 and 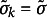 , for 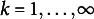 ; 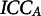 (32) is a generalization of its parametric version (23). The 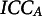 might reveal valuable information in inter-rater reliability or agreement analysis. For instance, when the ICC is used as an inter-rater reliability index (Erosheva et al., 2021; Martinková et al., 2023; Ten Hove et al., 2022), the 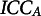 is the lower bound of the expected inter-rater reliability of a single rating.

In this work, we mainly focus on the population level 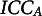 , but different ICC indices can be computed and compared under this framework by conditioning on different subjects or raters’ clusters.

We note that this class of ICCs generalizes those proposed by Lin et al. (2025) for multilevel data. As a consequence, propositions (1) and (2) hold for standard parametric multilevel models (Ten Hove et al., 2021, 2022). The BNP ICCs we propose account for heteroskedasticity across raters and naturally accommodate multiple clusters of both subjects and raters, whereas the standard ICCs commonly do not encompass these heterogeneous aspects.More details on these aspects are given in Appendix A.2.

## 4 Reduced model for one-way designs

One-way designs are common when raters’ identity is unknown and the systematic biases 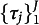 can not be identifiable. It might be seen as a limiting case in which each rater only scores one subject, i.e., 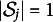 .

Some blocks of the model in Section 2.3 reduce as briefly presented below. Note that we model subjects’ true score 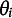 as in the main model (6) and (7).

*Modelling rating* 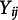 . We decompose the observed rating 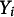 as (34)

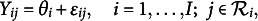

Here 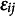 is the error of rater *j* in rating the subject *i* and it is the difference between the observed score 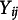 and the subject true score 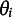 .

*Modelling raters’ error* 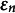 For each rating 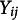 we assume that the rater’s error 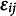 is drawn independently from a normal distribution with mean 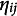 and variance 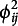 : (35)

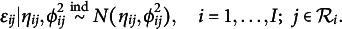

We specify a DP prior with concentration parameter 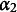 and base measure 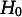 for the two-dimensional vector 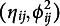 , for 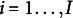 and 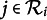 : (36)

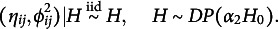

We assume 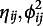 to be independent and choose 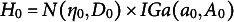 , where 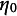 and 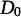 are mean and scale parameters, respectively. This formulation induces a DPM prior for raters’ error 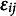 .

### 4.1 Identifiability and ICC

The moments of the error 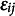 , 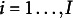 and 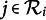 , are, respectively: (37)

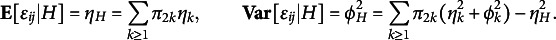

The centering strategy detailed in Section 2.5 is here used and a SC-DPM is here placed over 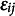 : (38)

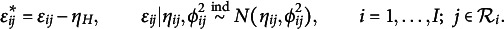

Under this parameter-expanded specification, the decomposition of rating 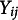 (34) becomes (39)

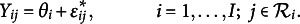

Given the location transformation in (38), the expectation of the residuals is zero: (40)

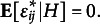

For the one-way designs, the exact general ICC might be consistently estimated.

Proposition 3. Given a random subject *i*, 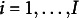 , independently scored by two random raters 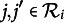 , 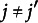 , the conditional correlation between the ratings 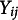 and 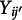 is (41)

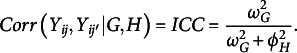

The proof is given in Appendix B. Conditioning on different clusters of subjects or raters and different ICC formulations lead to possible comparisons among clusters similar to the main model.

## 5 Posterior inference

The parameters of the DPs’ base measures (i.e., 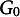 , 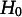 ) and the respective concentration parameters 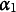 and 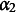 have to be assigned either a value or a hyperprior to complete the model specification and conduct posterior inference. This section outlines our choices about the hyperprior and the posterior computation. Several parameter specifications may be considered for the DP parameters (Ghosal & van der Vaart, 2017; Hjort et al., 2010) as they may be assigned a prior or fixed in advance. We placed a hyperprior on those parameters and let the data inform their parameters.

Under this model specification, the most natural choices to compute the posterior are conditional sampling schemes, such as Blocked Gibbs Sampling, which rely upon the approximate stick-breaking construction of the DP. They directly involve the prior in the sampling scheme avoiding its marginalization and accommodating hyperprior for the base measures (Ishwaran & James, 2001). They also come with further advantages, such as an improved mixing property, better interpretability of the mixture parameters (Gelman et al., 2014; Hjort et al., 2010) and the direct computation of the ICC. Indeed, avoiding the prior marginalization, the moments (16), (17), and (18) can be readily computed and plugged in the ICC formula (32).

However, tailored considerations have to be made in practical applications based on specific data features.

### 5.1 Hyperprior specification

Eliciting the concentrations ‘and base measures’ parameters has a role in controlling the posterior distribution over clustering (Gelman et al., 2014). Small values of the variance parameters of the base measures 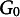 , and 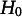 favor the clustering of subjects and raters, respectively, to different clusters. On the contrary, larger values of 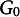 and 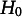 variances favor the allocation of different subjects and raters, respectively, to the same cluster.

We improve model flexibility by placing a prior on the base measures 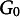 and 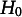 , and the concentration parameters 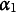 and 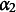 letting them be informed by the data. For the subjects’ true score base measure 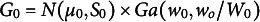 the following hyperpriors are specified: 

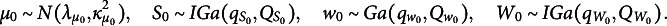

We let 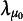 be the rating scale’s center value (e.g., 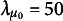 on a 1-100 rating scale), 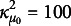 and the parameters 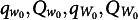 equal to 0.005. For the raters’ base measure 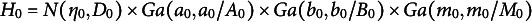 , the following hyperpriors are specified: 

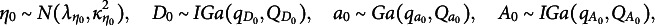




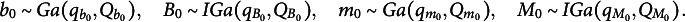

Where 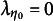 , 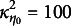 , and the other hyperparameters are fixed to 0.005. The concentration parameters 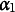 and 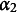 are assumed to follow, respectively, a gamma distribution: 

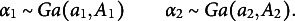

where 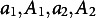 are fixed to 1. The values we fix for the hyperprior’s parameters are very common in literature and they are consistent with those proposed by many other studies on BNP models (e.g., Gelman et al., 2014; Heinzl et al., 2012; Mignemi et al., 2024; Paganin et al., 2023; Yang & Dunson, 2010).

### 5.2 Posterior computation

Since most of the parameters in the model have conjugate prior distributions, a Blocked Gibbs sampling algorithm was used for the posterior sampling (Ishwaran & James, 2001). No conjugate priors are available for the gamma’s shape parameters (e.g., 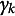 , 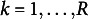 , 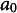 , 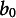 ), thus we approximate the full conditionals using a derivatives-matching procedure (D-M) which is involved as an additional sampling step within the MCMC. This method has several advantages over other sampling schemes (e.g., adaptive rejection sampling or Metropolis-Hasting) in terms of efficiency, flexibility, and convergence property (Miller, 2019). We use the same D-M algorithm introduced by Miller, 2019 to approximate the posterior of the gamma shape parameters of the base measures, i.e., 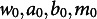 and a modified version for the parameters 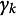 , 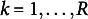 , since the parametrization (30) is adopted. We detail this adapted version of the D-M algorithm in the paragraph below and provide the complete Gibbs sampling in the Supplementary Material.

The notation on the independent allocation of subjects and rater to the corresponding clusters is introduced here. Let 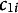 denote the cluster allocation of subject 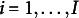 , with 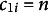 whenever 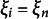 , 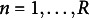 . Given the finite stick-breaking approximation detailed in Section 2.4, *R* is the maximum number of clusters. We indicate the set of all the subjects assigned to the *n*th cluster with 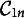 and with 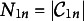 its cardinality. Accordingly, let 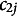 denote the cluster allocation of rater 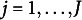 , such that 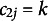 whenever 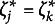 , 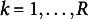 . The set of all the raters assigned to the *k*th cluster is denoted by 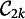 with 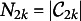 being its cardinality.

*Derivatives-matching procedure.* Since no conjugate priors are available for the gamma’s shape parameters 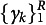 , we involve, for each of these parameters, a D-M procedure to find a gamma distribution that approximates the full conditional distribution of these parameters, when their prior is also a gamma distribution (Miller, 2019).

We aim to approximate 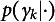 , i.e., the true full conditional density of 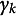 , 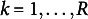 , by finding 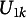 and 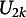 such that (42)

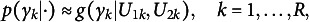

where 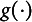 is a gamma density, 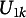 and 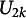 are shape and rate parameters, respectively. The algorithm aims to find 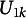 and 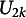 such that the first and the second derivatives of the corresponding log densities of 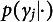 and 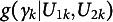 match at a point 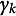 . Miller (2019) suggest to choose 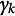 to be near the mean of 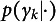 for computational convenience. The approximation is iteratively refined by matching derivatives at the current 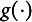 mean as shown by Algorithm 1. We adapt the algorithm to our proposal, more specifically we consider the model involving the shape constraint introduced in equation (30). When this constraint is not imposed, the original algorithm by Miller (2019) may be directly used.

We denote with 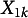 and 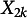 the sufficient statistics for 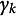 corresponding to the *k*th raters’ mixture component. For the implementation of the Algorithm 1 we set the convergence tolerance 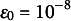 and the maximum number of iterations 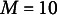 . Here 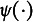 and 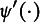 are the digamma and trigamma functions, respectively.

The parameters 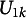 and 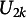 , returned by the algorithm, are used to update 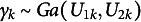 , 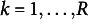 , through the MCMC sampling. The derivation of the algorithm is given in the Supplementary Material.

**Figure:**
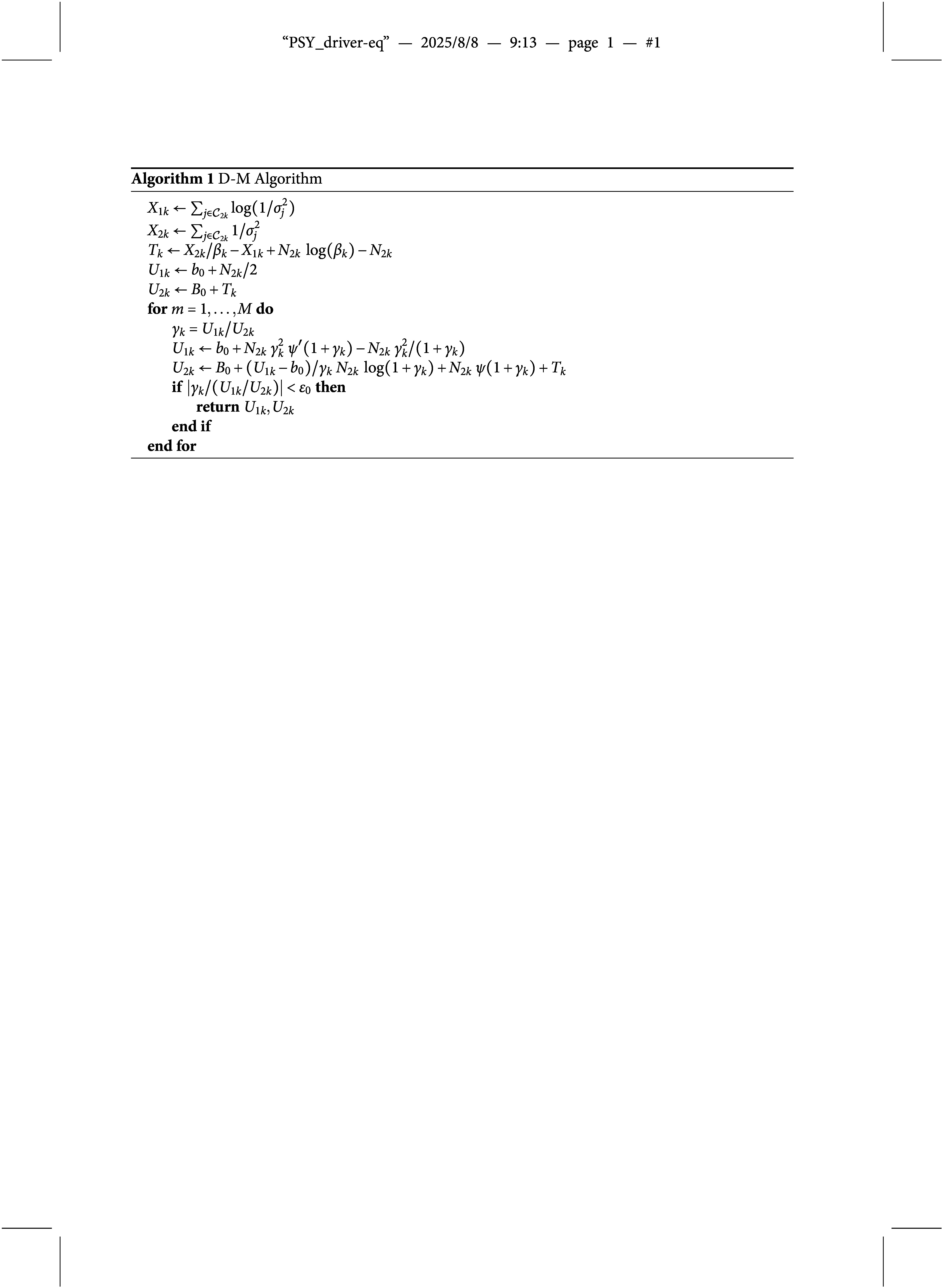

### 5.3 Post-processing procedures

*Semi-centered DPM processes.* The sampling scheme detailed in the Supplementary Material provides draws under the noncentered DPM model. However, as discussed in Section 2.5, it is not identifiable, and we need to post-process the MCMC samples to make inferences under the SC-DPM parameter-expanded model Yang & Dunson (2010). Since it is a semi-centered model that naturally constrains the raters’ systematic bias 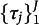 to have zero mean, a few location transformations are needed. After computing 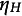 according to 17 for each iteration, the samples of 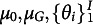 , and 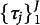 are computed: 

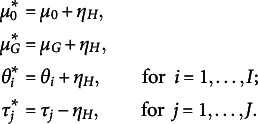

The first three are due to the location transformation of 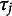 and have to be considered for inference purposes under the SC-DPM model.

*Posterior densities and clusters point estimates.* Each density equipped with a BNP prior might be monitored along the MCMC by a dense grid of equally spaced points (Gelman et al., 2014; Mignemi et al., 2024; Yang & Dunson, 2010). Each point of the grid is evaluated according to the mixture resulting from the finite stick-breaking approximation at each iteration. At the end of the MCMC, for each point of the grid posterior mean and credible interval might be computed, and as a by-product, the pointwise posterior distribution of the density might be represented.

The BNP model provides a posterior over the entire space of subjects’ and raters’ partitions, respectively. However, we can summarize these posteriors and determine the point estimates of these clustering structures by minimizing the respective variation of information (VI) loss functions. We refer to Wade & Ghahramani (2018) and Meilă (2007) for further details on VI and point estimates of probabilistic clustering.

As for every parameter of the model, we use the posterior distribution of the subjects’ specific parameters for inference purposes. Point estimates of the subjects’ true scores 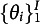 , such as the posterior mean (i.e., *expected a posteriori*, EAP) or the *maximum a posteriori* (i.e., MAP), might be used as official evaluations (i.e., final grades), and the posterior credible intervals as uncertainty quantification around those values. The 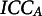 index (32) can be computed at each iteration of the MCMC to get its posterior distribution, which might be used for inference purposes.

*Computational details.* In the present work, both for the simulations and the real data analysis, similarly to previous works (e.g., Heinzl et al., 2012; Paganin et al., 2023), the number of iterations is fixed to 80,000 (with a thin factor of 60 due to memory constraints), discarding the first 20,000 as burn-in. We fix the maximum number of clusters to be 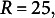 respectively, for subjects’ and raters’ DPM priors (Gelman et al., 2014). The package *mcclust.ext* (Wade, 2015) is used for the point estimate of the clustering structures based on the VI loss functions. We graphically check out trace plots for convergence and use the package *coda* for model diagnostics (De Iorio et al., 2023; Plummer et al., 2006). Convergence is also confirmed through multiple runs of the MCMC with different starting values^1^.

## 6 Simulation study

We perform a simulation study to compare the performance of the proposed models (BNP and a nested version) over the standard parametric one, highlighting the strength of our method. Concerning the *individual-specific level*, the three models are evaluated on the accuracy of the estimates of the individual-specific parameters they provide (i.e., how close 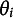 , 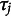 , 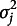 are to the respective true values). Regarding the *population level*, we compare the estimated population distribution of the subjects’ and raters’ features and evaluate the predictive performance of the three methods across different scenarios.

*BP model.* The first model is the Bayesian parametric one (BP model), which can be considered a reduction of the BNP model in which all the subjects and the raters are allocated to the same cluster, respectively, such that 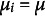 and 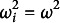 , for 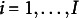 , and 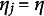 , 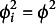 , 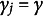 and 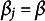 for 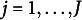 . This model might be obtained by fixing the maximum number of clusters 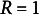 .

*BSP model.* The second model is the BSP one (BSP model), in which the normality assumption is relaxed for the subjects’ true score such that we model 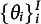 as detailed in Section 2.3, but we model the raters’ effects 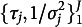 as in the parametric model (i.e., they are all assigned to the same cluster). This implies 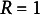 only for the rater-related DPM. Since in this model both *G* and *H* are degenerate on a mixture of only one component, we refer to the structural parameter as 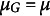 , 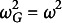 and 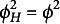 .

*BNP model.* The third model is the BNP model presented in Section 2.3 in which the normality assumption is relaxed both for subjects and raters. Under this model, subjects and raters are allowed to be, respectively, assigned to different clusters.

Three data-generative processes are set up with different clustering structures for subjects and raters. The densities of the subject’s true score and the rater’s effects are either unimodal, bimodal or multimodal. This allows us to assess the extent to which BNP priors might mitigate model misspecification and the BNP model reduces to the parametric one when the latter is properly specified; this setup is consistent with other works on BNP modeling in psychometrics (Paganin et al., 2023).

We keep some features of the generated data similar to the real data set analyzed in Section 7 (e.g., sample size, rating scale, ratings per subject), they are also comparable with those of other works on rating models (Bartoš & Martinková, 2024; Martinková et al., 2023). Additional simulation results on small sample size applications of our proposal are presented in the Supplementary Material.

### 6.1 Setting

We generate subjects’ ratings on a continuous scale, 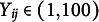 , the number of subjects 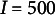 and raters 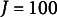 are fixed, whereas the number of ratings per subject and the true generative model vary across scenarios.

*Generative scenarios.* We manipulate the number of ratings per subject to be 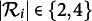 for 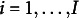 , since in many real contexts (e.g., education, peer review) it is common for the subjects to be rated only by two or few more independent raters (Zupanc & Štrumbelj, 2018).

Data are generated as specified by equations 5, 9, and one of the schemes below, according to the three different scenarios:

- **Unimodal:** Under this scenario, subjects’ true score and raters’ effects densities are unimodal: 

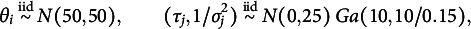

for 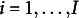 and 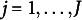 . This corresponds to the standard BP model in which subjects’ true scores are assumed to be i.i.d across subjects and raters’ effects are drawn jointly i.i.d. across raters.
- **Bimodal:** In this scenario, both subjects’ and raters’ populations are composed, respectively, of two different clusters: 

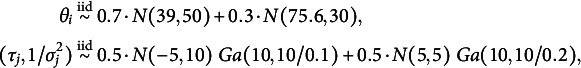

for 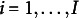 and 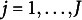 .
- **Multimodal:** Under this scenario, both subjects and raters are assigned, respectively, to three clusters: 

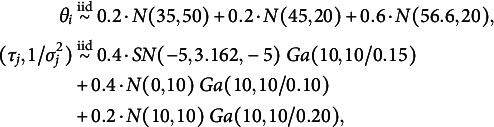

for 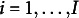 and 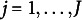 . Here 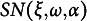 stands for the skew-normal distribution with location, scale and slant parameters, 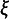 , 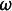 and 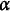 , respectively.

**Figure 3:**
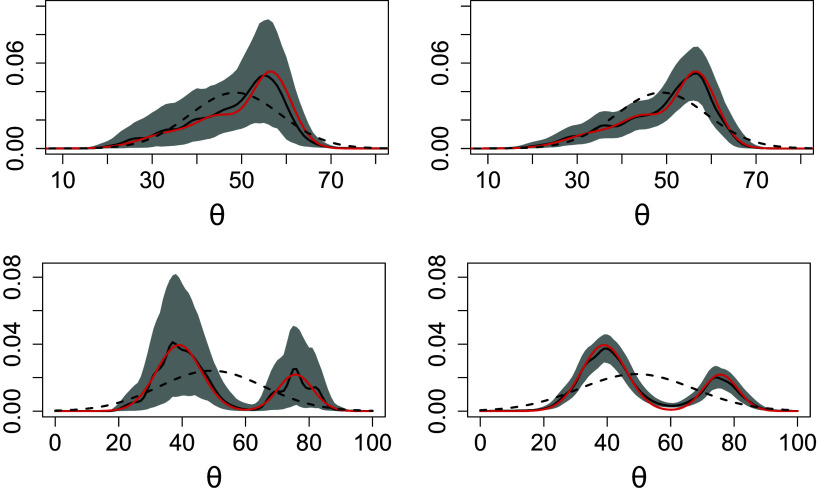
Average estimated density across 10 independent datasets under different scenarios. The columns indicate the cardinality of 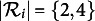 : left and right, respectively; the rows indicate *bimodal* or *multimodal* scenario: first and second row, respectively. The solid red lines indicate the true densities; the solid black line and the shaded grey area indicate, respectively, the point-wise mean and 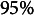 quantile-based credible intervals; the density implied by the BP model (black dotted lines).

**Figure 4:**
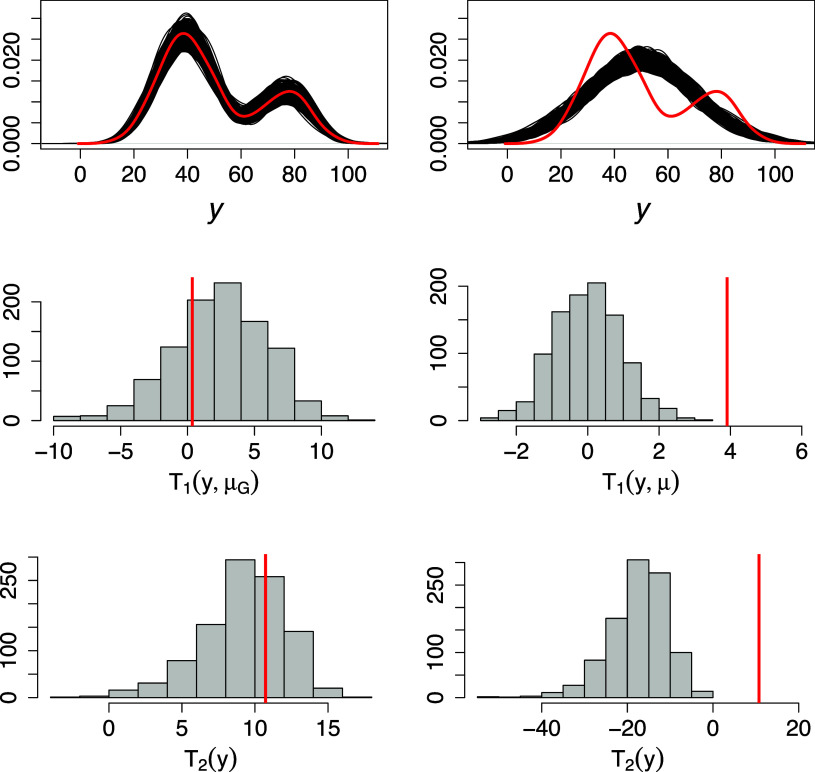
Top row: empirical distribution of the data (red solid line) and empirical distribution of replicated data (black solid lines) from the respective BNP and BP posterior distributions (left and right columns, respectively). Middle and bottom row: Test statistics computed on the data (red solid line) and histograms of those computed on replicated data.

**Figure C.1:**
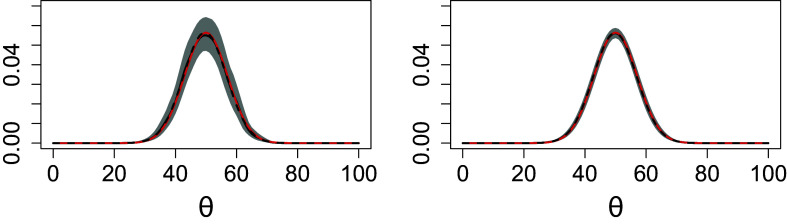
Average estimated density across 10 independent datasets under the *unimodal* scenario. The columns indicate the cardinality of 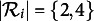 : left and right, respectively. The solid red lines indicate the true densities; the solid black line and the shaded grey area indicate, respectively, the point-wise mean and 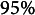 quantile-based credible intervals; the density implied by the BP model (black dotted lines).

These scenarios mimic three different levels of heterogeneity. From an interpretative point of view, in the first scenario, all the subjects’ true scores are concentrated around the center of the rating scale, and the raters are quite homogeneous in their severity and reliability. The heterogeneity of the subjects and the raters is only at the individual level since they are not nested with clusters. Under the second scenario, we introduce heterogeneity at the population level as both subjects and raters are assigned to different clusters, respectively. Here, we mimic the case in which subjects are clustered within two different levels of true score (e.g., low versus high proficiency level), and raters are either systematically slightly more lenient and reliable or more severe and less reliable. Under the third scenario, subjects and raters are assigned, respectively, to three poorly separated clusters. This results in a highly negatively skewed distribution for the subjects’ true score and a multimodal distribution for the raters’ systematic bias. Figures 3 and 4, Figure C.1 in Appendix C, and Figures 1, 2, and 3 in the Supplementary Material show the respective true densities and the empirical distributions of the generated ratings.

Ten independent data sets are generated under the six scenarios resulting from the 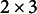 design, for each data set, the standard parametric (BP), the semi-parametric (BSP), and the nonparametric (BNP) models are fitted.

*Model recovery assessment.* Parameter recovery performance is assessed through the Root Mean Square Error (RMSE) and the Mean Absolute Error (MAE) computed, respectively, as the root mean square difference and the mean absolute difference between the posterior mean and the true value of the parameters across data sets. For the subject and raters specific parameters, i.e., 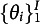 , 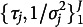 , RMSE, and MAE are average both across individuals and data sets.

For the sake of comparison across different scenarios, we report the standardized version of both indices (S-RMSE, S-MAE) for the structural parameters. More precisely, those related to 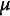 and 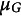 are divided by the mean value of the rating scale, i.e., 50; those regarding 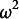 , 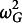 , 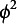 , 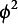 , 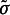 , 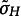 , and the 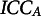 are divided by their true value.

The models’ performance in recovering the density distributions of individuals’ specific parameters is evaluated through visual inspection. We give an example of how different densities might lead to very different conclusions on the data generative process (Gelman et al., 2013; Paganin & de Valpine, 2024; Steinbakk & Storvik, 2009). Specifically, we draw new replications from the respective posterior predictive distributions and compare these samples to the original data. If the models capture relevant aspects of the data, they should look similar, and replications should not deviate systematically from the data. We measure discrepancy in central asymmetry through the statistic 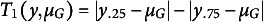 , where 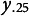 and 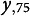 are the first and the third quartile, and in the left tail weight by the statistics 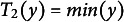 .

### 6.2 Results

Results from the simulation study suggest that our proposals (i.e., BSP and BNP) systematically improve the estimates of the individual-specific parameters across scenarios. However, the accuracy of these estimates is comparable under the *unimodal* scenarios across the three models. Meanwhile, the BSP and BNP models overcome, on average, the BP under the *bimodal* and *multimodal* scenarios in both conditions 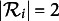 and 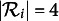 . As expected, the accuracy of subjects’ and raters’ specific parameters is higher in the conditions with a larger number of raters per subject 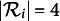 (Table 1). As indicated by 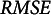 and 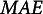 indices, on average, the estimates of subjects’ and raters’ specific parameters provided by all the models degrade from the *unimodal* to the *multimodal* scenario.

**Table 1:** RMSE and MAE of individuals parameters corresponding to BP, BSP and BNP models.

			Generative model					
			*Unimodal*		*Bimodal*		*Multimodal*	
			RMSE	MAE	RMSE	MAE	RMSE	MAE
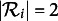	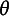	BP	**2.123**	1.686	2.346	1.846	2.497	2.009
		BSP	2.127	1.689	**2.308**	**1.822**	**2.347**	**1.889**
		BNP	**2.123**	**1.683**	2.327	1.841	2.439	1.961
	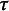	BP	1.404	1.102	1.575	1.208	1.892	1.566
		BSP	1.407	1.104	1.554	**1.206**	**1.700**	**1.387**
		BNP	**1.401**	**1.101**	**1.553**	1.212	1.774	1.460
	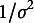	BP	0.070	0.060	0.092	0.076	0.085	0.070
		BSP	**0.069**	**0.059**	**0.071**	0.054	**0.066**	**0.050**
		BNP	0.071	**0.059**	**0.071**	**0.052**	**0.066**	**0.050**
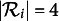	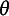	BP	1.442	1.154	1.512	1.192	1.817	1.471
		BSP	1.441	1.155	1.474	1.164	1.593	1.275
		BNP	**1.439**	**1.151**	**1.466**	**1.157**	**1.527**	**1.217**
	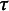	BP	0.860	0.688	0.920	0.726	1.384	1.157
		BSP	0.860	0.686	0.886	0.711	1.088	0.885
		BNP	**0.849**	**0.680**	**0.878**	**0.707**	**0.996**	**0.798**
	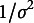	BP	0.037	0.029	0.054	0.042	**0.046**	0.036
		BSP	0.037	0.029	0.054	0.041	0.048	0.037
		BNP	0.037	0.029	**0.047**	**0.035**	0.047	**0.035**

*Note:* The bold text indicates the average, most accurate estimates.

**Table 2:** Standardized RMSE and standardized MAE of structural parameters corresponding to BP, BSP and BNP models.

			Generative model					
			*Unimodal*		*Bimodal*		*Multimodal*	
			S-RMSE	S-MAE	S-RMSE	S-MAE	S-RMSE	S-MAE
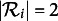	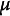	BP	0.015	0.014	0.020	0.017	0.029	0.028
	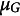	BSP	0.015	**0.012**	**0.014**	**0.010**	**0.022**	**0.021**
	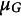	BNP	0.015	0.013	0.016	**0.010**	0.025	0.026
	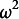	BP	**0.080**	**0.066**	0.284	0.284	**0.064**	**0.052**
	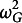	BSP	0.113	0.102	**0.040**	**0.036**	0.080	0.072
	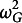	BNP	0.094	0.080	0.065	0.045	0.110	0.094
	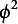	BP	0.979	0.110	2.343	2.341	0.273	0.239
	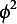	BSP	0.152	0.111	0.161	0.142	0.261	0.229
	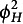	BNP	**0.134**	**0.103**	**0.112**	**0.084**	**0.195**	**0.173**
	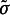	BP	0.244	0.242	**0.169**	**0.154**	0.225	0.221
	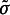	BSP	0.226	0.213	0.206	0.186	**0.097**	**0.081**
	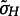	BNP	**0.223**	**0.209**	0.253	0.228	0.108	0.091
	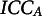	BP	0.002	0.002	0.001	0.001	0.023	0.023
		BSP	0.002	0.002	0.001	0.001	0.023	0.023
		BNP	0.002	0.002	0.001	0.001	0.023	0.23
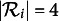	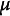	BP	0.013	0.011	0.022	0.019	0.027	0.022
	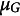	BSP	**0.012**	0.011	**0.017**	**0.014**	**0.018**	**0.015**
	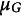	BNP	**0.012**	0.011	0.018	**0.014**	0.019	**0.015**
	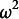	BP	**0.055**	**0.046**	0.281	0.281	**0.049**	**0.042**
	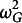	BSP	0.108	0.092	**0.046**	**0.043**	0.066	0.052
	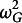	BNP	0.088	0.073	0.054	0.051	0.119	0.101
	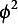	BP	0.994	0.110	2.319	2.317	0.275	0.258
	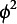	BSP	0.124	0.109	0.180	0.146	0.279	0.262
	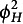	BNP	**0.119**	**0.105**	**0.132**	**0.095**	**0.209**	**0.188**
	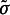	BP	**0.042**	**0.034**	**0.140**	0.130	**0.053**	**0.041**
	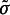	BSP	0.054	0.036	0.146	0.123	0.076	0.066
	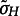	BNP	0.043	0.038	0.141	**0.114**	0.074	0.063
	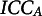	BP	0.002	0.002	0.001	0.001	0.023	0.023
		BSP	0.002	0.002	0.001	0.001	0.023	0.023
		BNP	0.002	0.002	0.001	0.001	0.023	**0.022**

*Note:* The bold text indicates the average, most accurate estimates.

Regarding the population parameter estimates, all the models provide overall similar estimates (Table 2). We observe the largest improvement of the BSP and BNP over the parametric model under the *bimodal* scenarios concerning subjects’ true score variance 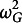 and raters’ systematic bias variance 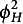 . However, in these cases, the BP model provides better estimates of the expected residual variance 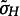 . As a result, these differences are not detectable in the 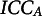 estimates and we observe equal accuracy for this index across the three models.

**Figure C.2:**
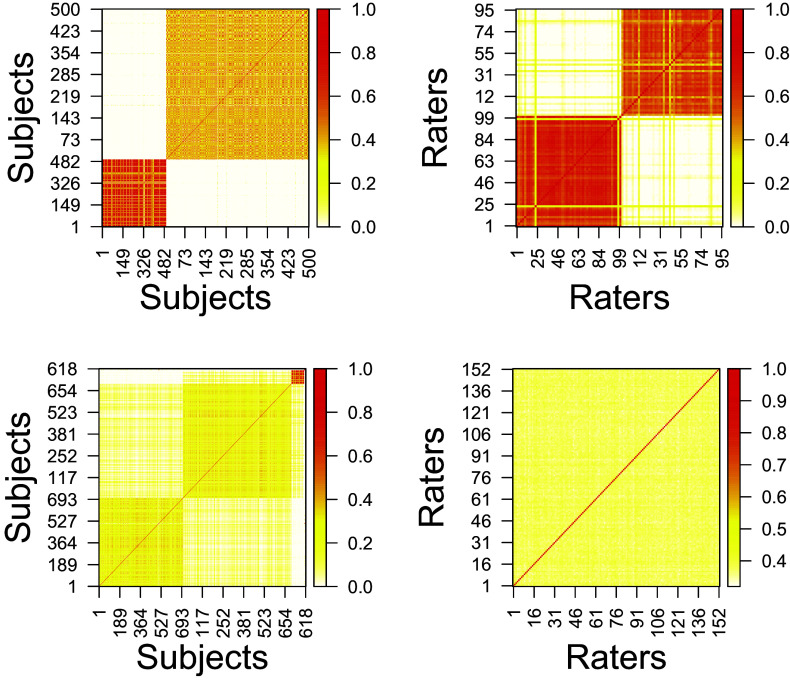
First row: examples of posterior similarity matrices for pairwise subject and raters allocation (left and right column, respectively). Second row: posterior similarity matrices for pairwise subject and raters allocation in real data analyzed in Section 7.

Figure 3 gives some examples of the estimated true score densities under the *bimodal* and *multimodal* scenarios; those under the *unimodal* scenario are reported in the Appendix C. The raters’ features density plots are shown in the Supplementary Material. The BNP model consistently estimates the respective densities under all the considered scenarios. The most prominent improvement of our proposals over the parametric model is observed under the heterogeneous scenarios. Accurate estimates of the densities are also provided under the extreme case of 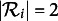 , that is, when each subject is rated by only two independent raters. Nonetheless, we note that the uncertainty about the densities is reduced when subjects are rated by a larger number of raters (i.e., 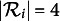 ). This reduction mostly regards the subjects’ true score densities across all the scenarios. Our proposals capture the latent clustering structures of both subjects and raters as displayed by the posterior similarity matrices in Figure C.2 in Appendix C. The entries of these matrices are the pairwise probability that two entries (e.g., subjects or raters) are clustered together. The clustering structure implied by the generative process under the *bimodal* scenario is readily recognized by the graphical inspection.

The BNP model effectively captures relevant latent aspects of the data, such as deviations from normality both in the center and in the tails of the distributions across all the scenarios. As a by-product, the replications drawn from the posterior predictive distribution of the BNP model are remarkably more plausible than those generated under the BP model. As shown in Figure 4, the normality assumptions made in the latter model restrict the shapes of the distributions for subjects’ and raters’ features. As a result, when these assumptions are violated, any inferences about the data-generating process might be misleading and unreliable. Replications under the BP model are far from the data both in the centre and on the tails of the distribution, as suggested by the statistics 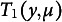 and 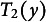 in Figure 4.

The improvement of our method over the parametric one is more prominent when the design is balanced (e.g., fully crossed designs) and the samples of subjects and raters are smaller. We present these results in the Supplementary Material.

## 7 Application on large-scale essay assessment

**Figure 5:**
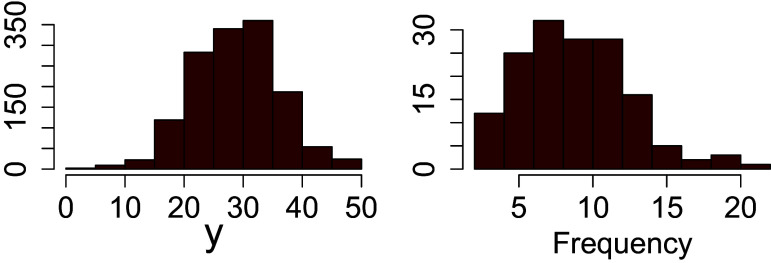
The empirical distribution of ratings and the frequency of students per teacher are reported at left and right, respectively.

We analyze the *Matura* data set from Zupanc & Štrumbelj (2018) as an illustrative example. The data come from a large-scale essay assessment conducted by the National Examination Centre in upper secondary schools in Slovenia during the nationwide external examination. Each student received a holistic grade on a 1-50 rating scale by two independent teachers. We considered a random sample of 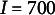 students out of the 6995 who were examined during the spring term argumentative essays for the year 2014. A sample of 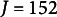 teachers were involved who graded, on average, 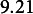 students, with a minimum of 2 and a maximum of 21 (see Figure 5). The observed ratings ranged from 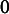 to 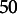 , with a mean of 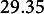 , a skewness of 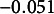 , and a kurtosis of 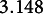 (see Figure 5). More details about the assessment procedure might be found in Zupanc & Štrumbelj (2018).

*Model comparison.* The three different models detailed in Section 6, i.e., the parametric (BP) model, the semiparametric (BSP) model, and the nonparametric (BNP) model, were fitted to these data and compared on their out-of-sample prediction accuracy. The Watanabe–Akaike information criterion (WAIC) was used for this purpose.

This is a fully Bayesian approach for estimating the out-of-sample expectation, which relies on the computed log pointwise posterior predictive density and on a penalty term correction for the effective number of parameters to prevent overfitting (Gelman et al., 2014). The respective WAIC formulas are provided in the Supplementary Material.

### 7.1 Results

The total computational elapsed time for the BP, BSP, and BNP models was 180, 300, and 355 minutes, respectively. No convergence or mixing issues emerged from the graphical inspections of the MCMCs and diagnostics from *CODA* package (Plummer et al., 2006); further details and examples of trace plots are given in the Supplementary Material. Table 3 shows the WAIC indices for each fitted model and shows that the selection procedure indicates that the BNP model best fits the data and overcomes the others in predicting out-of-sample ratings. These results are consistent with the additional hold-out validation procedure presented in the Supplementary Material. Based on the model comparison procedure, we focus on the results from the BNP model.

**Table 3:** The WAIC is reported for each of the fitted models: BNP, BP, and BSP; the pairwise WAIC difference ( 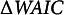 ) between the model with the best fit and each other is reported

Fitted model	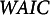	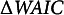
BNP model	56267.43	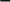
BSP model	67159.21	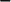 10891.78
BP model	168701.8	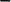 112434.4

**Table 4:** Posterior mean and 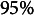 quantile-based credible intervals of the estimated structural parameters of the BNP model are reported

		Posterior mean	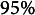 Credible interval
Subjects’ parameters	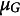	29.126	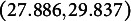
	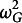	32.702	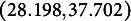
	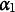	4.053	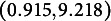
Raters’ parameters	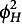	5.465	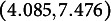
	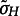	13.913	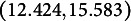
	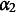	1.839	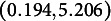
	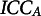	0.627	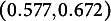

**Figure 6:**
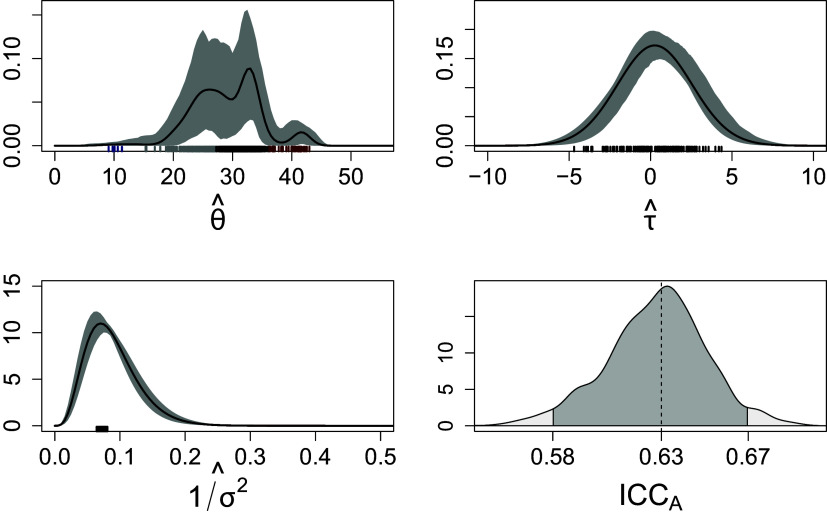
The estimated densities of the subject’s true score 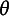 , rater’s systematic bias 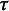 and the residual term 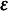 are reported; the black solid lines and the shade grey areas indicate the pointwise posterior mean and 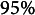 quantile-based credible intervals of the respective densities. Bottom-right figure shows the posterior distribution of the 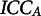 , the black solid and dotted lines indicate, respectively, the 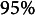 credible interval and the posterior mean. The rugs at the margins of the first three figures indicate the clustering of individuals.

The posterior expectation of student ability mean 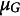 and variance 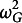 population parameters are 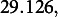 and 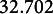 , respectively (Table 4). The respective narrow credible intervals suggest low uncertainty about these values. As expected from Antoniak (1974), the posterior values of the concentration parameters 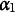 and 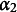 are proportional to the respective sample sizes and larger for the former. Details of the posterior values of base measures’ parameters are reported in the Supplementary Material. The posterior expectation of raters’ systematic bias variance 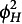 and reliability 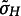 are, respectively, 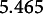 and 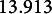 . The corresponding credible intervals suggest low uncertainty around these values (Table 4).

Figure 6 gives the graphical representation of the respective estimated densities. The multimodal distribution of student ability 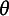 implies heterogeneity among student abilities and points to the presence of multiple sub-populations. The variance in ratings is broadly due to students’ ability, despite the variability of raters’ systematic bias and reliability. Regarding the clustering structure of subjects and raters, the posterior similarity matrix, reported in Figure C.2 in Appendix C, suggests the presence of some latent partition of subjects, whereas no evidence of raters’ clusters emerged from the posterior. This is coherent with the clusters’ point estimate based on the variation of information (VI) loss function, which indicates four clusters for the subjects and one cluster for the raters. We render this result in Figure 6 through rugs of different colors at the margin of the density plots; these values indicate the posterior mean of each subject and rater specific parameter. It is worth noting that we observe a cluster of subjects whose proficiency level is remarkably lower than the others, and another cluster in which subjects’ performance is slightly superior than the others (Figure 6, upper-left; blue and brown rugs, respectively). These subjects might benefit from more personalized and specialized educational pathways. The posterior distribution of the 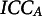 with mean and credible intervals, respectively, equal to 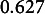 and 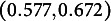 , suggests a moderate inter-rater reliability; Figure 6 shows the posterior distribution of this index. Since 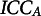 might be interpreted as the lower bound of the expected inter-rater reliability of a single rating, poor levels of reliability can be excluded (Koo & Li, 2016). However, this result is coherent with the findings of the original study by Zupanc & Štrumbelj (2018), where raters’ variability and reliability have a substantial effect on ratings. Aggregate or average ratings over different teachers might mitigate inter-rater reliability issues (Erosheva et al., 2021).

## 8 Coarsened ratings extension

Ratings data might be arbitrarily coarsened into a small number of ordered categories (Goel & Thakor, 2015; Harbaugh & Rasmusen, 2018; Peeters, 2015; van Praag et al., 2025). As a result, continuous ratings that fall between two consecutive cut-offs are collapsed into the same ordered category, and fine-grained distinctions between individual scores are missing (Ho & Reardon, 2012; Reardon et al., 2017). The available ratings are ordinal in these cases, and the rating model proposed in Section 2.3 has to be modified accordingly.

We leverage the underlying response variable formulation to extend the model to the ordinal case and consider the data coarsening mechanism (Agresti, 2015; Albert & Chib, 1993; Bartholomew et al., 2011; Cao et al., 2010; Nelson & Edwards, 2015). Our proposal might be seen as a BNP extension of the heteroscedastic ordered probit (HETOP; Lockwood et al., 2018). We specify the cumulative density function of the standard normal 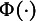 as a link function, which implies that we only need to modify the equation (5). This extension might readily adapt to the One-Way designs presented in Section 4.

We note that coarse and ordinal ratings might be rather different. In the first case, the categories are consecutive intervals of a continuous rating scale, which is not the case for ordinal ratings. Here, we propose the HETOP specification as a possible straightforward extension of the main model for coarsened ratings and leave more advantageous formulations for ordinal data for future investigations.

### 8.1 Categorical modeling

*Modeling rating* 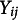 We assume that the observed ordinal rating 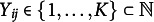 is generated by an underlying unobserved normally distributed variable 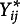 (Jöreskog & Moustaki, 2001) and that we observe 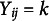 if 
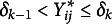
 ; 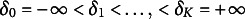 are ordered thresholds over the underlying response variable distribution and are equal across raters. The underlying variable 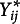 might be interpreted as a latent rating or the original continuous rating before the coarsening procedure. The conditional probability that 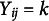 is (43)

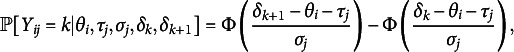

for 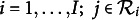 . Additional considerations on the interpretation of 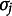 under this formulation are given in the Supplementary Material.

*Identifiability issues.* Under this parametrization, we need to put additional constraints for identifiability purposes since the underlying response variables’ mean and variance are freely estimated (DeYoreo & Kottas, 2018; Kottas et al., 2005). Two thresholds (e.g., 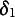 , 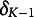 as proposed by Song et al., 2013) have to be fixed in advance, as it is common in multi-group analysis (Lockwood et al., 2018). From a statistical perspective, we note that each rater might be seen as a group of observations (Papaspiliopoulos et al., 2023). Moreover, an SC-DPM prior has to be placed on the subject’s true score 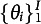 to fix their mean and resolve identifiability issues (Gelman et al., 2014), as a by-product under the parameter-expanded specification, equation (43) becomes (44)

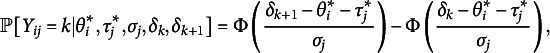

for 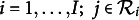 . Whenever 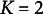 , i.e., dichotomous rating scale, 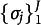 can not be identified and need to be fixed in advance, e.g., 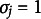 , 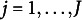 , which implies assuming raters to be equally reliable (Lockwood et al., 2018).

*Generalized ICCs.* Under this model specification, the ICCs computed according to propositions 1 and 2 are generalized ICCs that indicate the polychoric correlation between two latent ratings (Jöreskog, 1994; Uebersax, 1993). For instance, proposition 1 implies here: (45)

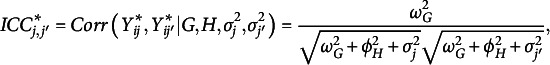

where 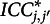 indicates the conditional pairwise polychoric correlation between the latent ratings given by raters 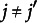 to subject *i*. Similar considerations might be extended to propositions 2 and 3. As a by-product, the 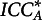 is the lower bound of the expected polychoric correlation between the latent ratings 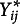 and 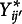 , with 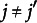 : (46)

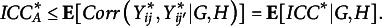



### 8.2 Posterior computation

A data augmentation procedure may simulate the underlying response variables (Albert & Chib, 1993). The underlying continuous ratings 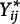 , 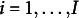 , 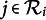 are sampled: 

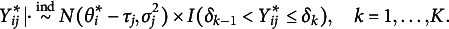

Here 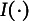 is an indicator function. Following Albert & Chib (1993) the conditional posterior distribution of the 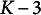 freely estimated thresholds,e.g., 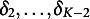 might be seen to be uniform on the respective intervals: 

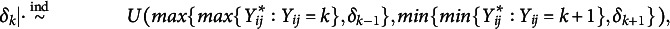

here 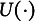 stands for uniform distribution.

All the other parameters are updated according to the posterior sampling scheme detailed in Section 3.1 of Supplementary Material and the post-process transformation outlined in Section 5.3 needs to take into account the double-centering. After computing 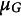 and 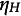 according to 16 and 17 for each iteration, the samples of 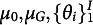 , and 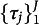 are computed as follows: 

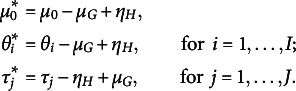



### 8.3 Generated and real coarsened ratings analysis

In this section, we present the analysis of real and generated coarsened ratings and compare the results with those presented in Sections 6 and 7. For the real data, we deliberately coarsened the original continuous ratings analyzed in Section 7 into 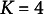 ordered categories according to the following cutoffs: 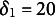 , 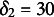 , 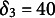 . The fit of the BP, BSP, and BNP models to the data are compared according to the WAIC for ordered data discussed in the Supplementary Material.

We performed a simulation study to assess the accuracy of the BNP and the BP versions for ordered ratings. More specifically, the same data sets generated under the *bimodal* scenarios in Section 6 are coarsened and considered for this study. We coarse these ratings into 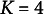 ordered categories according to three consecutive cutoffs: 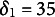 , 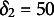 , 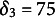 . The same parameter recovery assessment procedure detailed in Section 6 is consistently used here.

**Table 5:** RMSE and MAE of individuals parameters across *bimodal* scenarios with coarsened ratings

		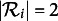		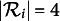	
		RMSE	MAE	RMSE	MAE
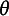	BP	6.333	5.151	4.995	4.016
	BNP	**4.846**	**3.802**	**3.677**	**2.883**
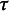	BP	3.197	2.532	2.002	1.586
	BNP	**2.896**	**2.278**	**1.832**	**1.437**
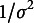	BP	0.195	0.184	**0.065**	**0.054**
	BNP	**0.104**	**0.080**	0.097	0.074

*Note:* The bold text indicates the average, most accurate estimates.

**Table 6:** The WAIC is reported for each of the fitted models: BNP, BP, and BSP; the pairwise WAIC difference ( 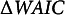 ) between the model with the best fit and each other is reported

Fitted model	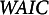	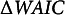
BNP model	3798.11	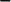
BP model	3815.65	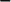 17.54
BSP model	3897.22	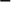 99.11

In real context, the cutoffs of the coarsening procedure are generally known since the continuous rating scale is deliberately broken down into a small number of consecutive intervals and raters are explicitly asked to coarse their ratings accordingly (Peeters, 2015; van Praag et al., 2025). For example, on a 1–100 continuous scale, they might be asked to indicate which of the following intervals each subject’s score falls into: (1–25), (25–50), (50,75) or (75,100). On the contrary, when ratings are directly given on an ordinal scale, the categories’ labels are not necessarily associated with any continuous scale intervals (e.g., “poor”, “acceptable”, “good”, “very good”). In these scenarios, we consider the observed ordered ratings as coarsened representations of underlying continuous values according to some unknown consecutive cutoffs. In the first case, this coarsening process is factual; in the second, it is merely assumed. However, since in both cases at least two cutoffs need to be fixed for identification purposes, we decide to fix 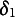 and 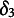 to the true values and let the model estimate 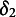 , both for real and generated data.

**Figure 7:**
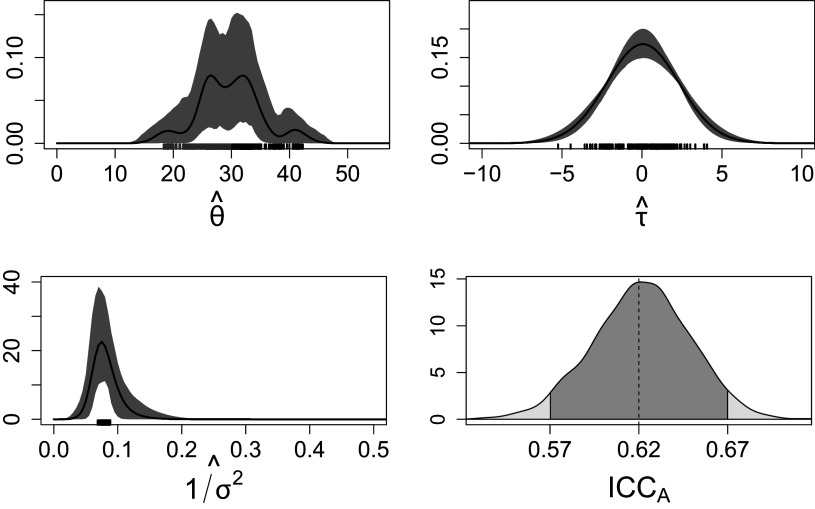
The estimated densities of the subject’s true score 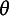 , rater’s systematic bias 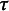 and the residual term 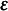 are reported; the black solid lines and the shade grey areas indicate the pointwise posterior mean and 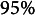 quantile-based credible intervals of the respective densities. Bottom-right figure shows the posterior distribution of the 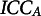 , the black solid and dotted lines indicate, respectively, the 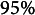 credible interval and the posterior mean. The rugs at the margins of the first three figures indicate the clustering of individuals.

*Results.* The total computational elapsed times for the BP, BSP, and BNP models were roughly similar to those of previous Sections. Upon graphical inspections of the MCMC chains and diagnostics, no convergence or mixing issues emerged for both generated and real data. Table 6 gives the WAIC indices for each fitted model and suggests that the BNP model provides the best fit to the data. Based on this model comparison procedure, we focus on the results from the BNP model. As shown in Table 7, the estimates are equivalent to those obtained under the continuous BNP model presented in Section 7. We note that the only notable difference concerns the point estimate of the subjects’ clustering structure (Figure 7). In this case, they are clustered into two (instead of four) subjects’ groups.

**Table 7:** Posterior mean and 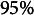 quantile-based credible intervals of the estimated structural parameters of the BNP model are reported

		Posterior mean	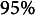 credible interval
	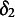	29.671	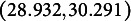
Subjects’ parameters	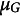	29.678	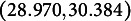
	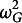	30.513	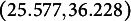
	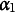	4.174	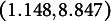
Raters’ parameters	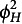	5.958	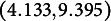
	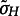	13.1080	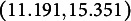
	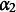	1.911	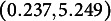
	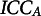	0.627	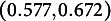

Results from generated data suggest that the BNP model provides more accurate estimates of subjects’ and raters’ specific parameters and overcomes the BP model. The only exception is observed for the rater-specific reliability parameter 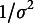 under the scenario 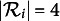 ; here, the BP model overcomes our proposal. Under the standard parametric model, we only have two population parameters 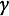 and 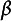 (i.e., 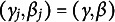 , for 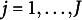 ) and, as a consequence, more information is available for their estimation. This might result in a faster accuracy improvement of this model for this set of parameters as the ratio of students per rater increases. The comparison between the 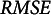 and the 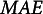 of Tables 1 and 5 suggests that the estimates of both the BP and BNP models degrade with coarse data. The same trend emerged regarding the structural parameters and the densities; we report these results in the Supplementary Material.

## 9 Concluding remarks

A flexible BNP framework is proposed for the analysis of holistic rating data . We adopt the two-way unbalanced design as a general setting (McGraw & Wong, 1996) which allows us to relate our proposal to other existing models (e.g., cross-classified or crossed random effects models, multilevel models, IRT-based rating models). We specify a measurement model to jointly estimate the subject’s latent quality (e.g., student’s proficiency) and the rater’s features (i.e., severity and consistency). Our proposal may be suitable both for balanced (i.e., when all raters score each subject; Nelson & Edwards, 2010, 2015) and unbalanced designs (i.e., when a subset of raters scores each subject; Martinková et al., 2023; Ten Hove et al., 2022). This method aims to capture latent heterogeneity among subjects and raters with the stochastic clustering induced by the DPM placed over their effects. This allows us to relax the common distributional assumptions on the respective parameters, preventing model misspecification issues (Antonelli et al., 2016; Walker & Gutiérrez-Peña, 2007).

Results from the simulation study highlight the flexibility of our proposal, which provides accurate estimates across different scenarios. Exploiting the DPM prior, the respective densities of the students’ and raters’ effects are consistently estimated both when the normality assumption holds and when it is violated. Our method provides a more prominent improvement in small sample sizes and with coarse data. Our proposal provides the best fit to the real data, both for continuous and coarse ratings, compared to the parametric competitor. Nonetheless, the accuracy of the estimates with coarse ratings might be a concern when subjects are only rated by a very small number of raters and the estimated true scores are used, for instance, for selection purposes or as official grades. The theoretical results presented in Section 3 are employed to make inferences about the inter-rater reliability of the single ratings.

The relatively long computational times of the MCMC chains might be prohibitive if used for repeated or massive scoring procedures. In such cases, if one is interested in capturing systematic heterogeneity among subjects or raters, any formulation of a mixture model (parametric or nonparametric) might be computationally cumbersome. In contrast, if this is not the focus of the analysis, the parametric model might be a computationally faster solution.

Under our model, rater’s systematic bias and reliability are assumed to be independent conditional on the parameters of the cluster; additionally, the reliability of the raters is assumed to be independent of their specific workload 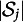 (i.e., the cardinality of the subset of subjects the rater has to evaluate). These assumptions might be unrealistic in some real contexts, and they might be relaxed under more general model specifications. For example, a multivariate distribution might be specified as a base measure 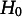 to account for the correlation between the rater’s features, and the rater-specific workload 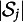 might be modeled as a random variable correlated to the rater’s features. Furthermore, because the measurement model includes raters’ effects only as an additive component, all raters are assumed to have the same ability to discriminate between subjects with different latent true scores. This assumption might be relaxed by specifying an additional rater-specific multiplicative effect for the subject’s true score, similar to the GMFRMs (Uto & Ueno, 2016).

The model detailed in Section 2.3 might be further extended to account for multidimensional ratings, i.e., when subjects are rated on multiple items. Under this three-way design, item parameters might be identified under some general conditions, and the model might extend Paganin et al., 2023, or Karabatsos & Walker, 2009 to account for raters’ characteristics. Further BNP generalizations of the existing rating models, e.g., GMFRMs, (e.g., Uto & Ueno, 2016; Uto et al., 2024) HRMs (e.g., Casabianca et al., 2015; DeCarlo et al., 2011; Molenaar et al., 2021) or Trifactor Models (e.g., Shin et al., 2019; Soland & Kuhfeld, 2022) are left for future investigations.

The effect of covariate and contextual factors might be incorporated in the structural models 6, 8, or 9 if additional information on subjects or raters is available. This extension might relate our model to Explanatory Response Models (Kim & Wilson, 2020; Wilson & De Boeck, 2004) and be a BNP generalization of those methods. According to the data structure, more complex hierarchical priors might be placed over the subjects’ true scores, such as hierarchical (Paisley et al., 2014; Teh et al., 2004), nested DPMs (Gelman et al., 2014; Hjort et al., 2010; Rodriguez et al., 2008) or hidden hierarchical DPMs recently introduced by (Lijoi et al., 2023) which overcomes some flaws of the previous ones. Stochastic Approximations of the DPM might be further considered for the stick-breaking constructions avoiding a maximum number of clusters (Arbel et al., 2019).

Our method might provide practitioners with valuable insights about the subjects’ and raters’ specific features along with the respective clustering structures. This information might be used to great advantage of individualized teaching programs (Coates, 2025) and might improve the matching procedure between subjects in peer teaching activities (Stigmar, 2016). Our theoretical finding and computational solution might enhance the analysis of rating data and contribute novel knowledge about the rating process.

## Supporting information

Mignemi and Manolopoulou supplementary material Mignemi and Manolopoulou supplementary material

## Footnotes

1 CPU configuration: 12th Gen Intel(R) Core(TM) i9 12900H.

## Supplementary material

The supplementary material for this article can be found at https://doi.org/10.1017/psy.2025.10035.

## A Further extensions

In this section, we present some model extensions for more flexible clustering and complex hierarchical structures. We briefly detail alternative discrete priors that generalize the Dirichlet Process, and provide a more general framework accounting for multiple source of variability.

### A.1 DP generalizations

Following the notation in Section 2.2, given  the number of different unique values  generated by *p* increase asymptotically at a logarithmic rate, with  a.s. for  . Alternative priors might be specified over *p* which overcome this issue and allow for a more flexible prior specification on the number of clusters. More general specifications of  are briefly presented below.

Our proposal might readily encompass these priors, and since they all share the stick-breaking representation presented in Section 14, the  estimation and the Semi-centered identifiability procedure still hold for these cases.

#### A.1.1 Mixture of Pitman–Yor process

One of the most common generalizations of the Dirichlet Process is the Pitman–Yor Process  , indexed by a discount parameter  , a concentration parameter  , and a base measure  . This is also termed the two-parameter Poisson DP. For instance, we can place the  as a prior over the subject random measure  , which might be represented as

Under this specification, the number of observed clusters  out of a sample of *I* subjects increase asymptotically at a rate  , with  as  . Here  is a limiting random variable with a probability distribution depending on *d* and  and a positive density on  . For  , we recover the  , whereas for larger values of *d*, the rate of increase of  is faster. The discount parameter *d* might be interpreted as the proportion of small clusters that will be observed out of a sample of *I* subjects. Indeed, this parameter plays a double role in the clustering behavior of the model. The higher values of *d* imply a *reinforcement mechanism* that favors the allocation of a subject to the larger clusters (the “rich-get-richer” property) and, at the same time, a higher probability of being assigned to a new cluster. This is clear from  , for  , which suggests that the decay of the cluster sizes is governed by a power law.

#### A.1.2 Mixture of Normalized Generalized Gamma process

We can alternatively specify a Normalized Generalized Gamma (NGG) process as a prior for  (Brix, 1999; Lijoi et al., 2007). This distribution is characterized by  ,  , and a base measure  . Following the previous example, we can consider the subject random measure to be distributed according to an NGG,  . It might be represented as

where  are points of a generalized gamma process with parameters  ,  , and  (Brix, 1999). For  we recover the DP. See Ghosal & van der Vaart (2017) for the correspondence between the PY and NGG processes. The interpretation of the parameters  and *d*, and the comments on the power law tails behavior of the PY process might be readily applied to the NGG process.

In educational rating contexts, the PY and the NGG processes might be preferred to the DP when the interest is to identify a few large clusters of subjects with similar proficiency levels and subjects who might need more *one-on-one* or personalized teaching. We refer to De Blasi et al. (2015); Hjort et al. (2010) and Ishwaran & James (2001) for a broader treatment of this class of priors.

### A.2 Multiple ways unbalanced designs

We present here an extension of our proposal for multiple-way unbalanced designs. Without loss of generality, we consider a three-way design in which ratings are additionally nested within an independent factor. It is the case where subjects are rated over different tasks (Ten Hove et al., 2022) or across different occasions (Lin et al., 2025).

Consider a subject  , whose attribute is independently scored by a random subset of raters  on a continuous rating scale across different tasks  . We assume that the observed rating  depends independently on subject *i*, rater  and task  . We let the residual part, that is the difference between the true and the observed score, depend on rater *j*’s effects, i.e. systematic bias and reliability. We specify the following decomposition of rating  :

(47)

for  ,  and  . Here  is the true score of subject *i*,  is the systematic bias of rater *j*,  is the average easiness of task *d*,  is the average bias of rater *j* in scoring subject *i*,  is the easiness of task *d* for subject *i*,  is the bias of rater *j* in scoring subject *i* in task *d* and  is a residual term following a zero-mean normal distribution with variance  as in the two-way unbalanced design. All the parameters in (47) are assumed to be mutually independent.

We extend the model presented in Section 2.3 by placing a prior over the new set of parameters. They might be equivalently equipped with parametric or nonparametric priors. In the latter case, the same modelling strategy as described for  in Section 2.3 and 2.4 might be freely adopted for any of the new parameters.

The ICCs class for these multiple-way models might be derived straightforwardly as in the two-way design setting and following the rationale detailed in Lin et al. (2025). If nonparametric priors are placed over the new set of parameters, the variance of their respective mixture distribution might be estimated as indicated in Section 2.4. As for the two-way case, the BNP ICCs class for these models is a generalization of most of those proposed for multilevel data (e.g., Lin et al., 2025; Ten Hove et al., 2021, 2022), since the residuals’ heteroschedasticity and the flexible mixture distribution of the parameters. As a consequence, when parametric priors are placed over the parameters in (47) and given  , for  , the standard ICCs in Lin et al. (2025) are recovered.

## B Proofs

### B.1 Proof of Proposition 1

Proof. Let  and  be the ratings given by two random raters  , to a random subject *i*, for  :

Assuming mutual independence between the terms of the decomposition:

and the conditional covariance between the two ratings is

The correlation between the ratings is

### B.2 Proof of statement (i) of Proposition 2

Proof. Let  and  be the ratings given by two random raters  , satisfying  to a random subject *i*,  :

Assuming mutual independence between the terms of the decomposition:

and the conditional covariance between the two ratings is

The correlation between the ratings is

### B.3 Proof of statement (ii) of Proposition 2

Proof. Let us consider the function  which, conditional on *G* and *H*, is a convex function of the random variable  :

(48)

. Let  be the  function of the expected value of  :

(49)

Note that  for  ,  . It readily follows from the conditional Jensen’s Inequality that

(50)

Since for brevity we define  and  :

(51)

where  ,  and  . That is the expected correlation between two independent ratings given to a random subject.

### B.4 Proof of Proposition 3

Proof. Let  and  be the ratings given by  , to a random subject *i*,  :

Assuming mutual independence between the terms of the decomposition:

and the conditional covariance between the two ratings is

The correlation between the ratings is

Since the conditional variance of ratings is equal across subjects  for  , the ICC is unique for all the subjects.
