# Bayesian Nonparametric Models for Multiple Raters: a General Statistical Framework - ERRATUM

**DOI:** 10.1017/psy.2025.10062

**Published:** 2025-12-01

**Authors:** Giuseppe Mignemi, Ioanna Manolopoulou

**Affiliations:** 1 Bocconi Institute for Data Science and Analytics, Bocconi University, Milan, Italy; 2 Statistics Department, University College London, Bloomsbury, UK

The above article was published with an error in the first equations in Section 8.2 on page 1469. The equation should have read as follows:

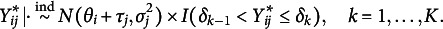



## References

[r1] Mignemi, G. , & Manolopoulou, I. (2025). Bayesian Nonparametric Models for Multiple Raters: A General Statistical Framework. Psychometrika. 90(4), 1445–1480. 10.1017/psy.2025.10035 40785396 PMC12660027

