# Supplementary material for: Bayesian Nonparametric Models for Multiple Raters: A General Statistical Framework
Source: Psychometrika. 2025 Aug 11;90(4):1445–80. doi: 10.1017/psy.2025.10035 (PMC12660027; doi:10.1017/psy.2025.10035)
Supplement: Mignemi and Manolopoulou supplementary material [file S0033312325100355sup001.zip › S0033312325100355sup001.pdf]

Bayesian Nonparametric Models for Multiple Raters: a General Statistical Framework
Supplementary Materials

1 Small Sample Simulation Study

Rating procedures are commonly used to assess inter-rater agreement on the same set of subjects (**???**). Data coming from these settings are characterized by a small set of raters who independently rate the same (small) set of subjects. We performed a small simulation study to assess the performance of our model for these cases and compare it to the parametric version.
We consider here a setting with a small set of raters which can not be considered a representative sample of the population of raters. For this reason, we propose the semi-parametric version of our proposal (the BSP) as an alternative to the standard parametric model (BP) for these cases. As a consequence, we assume that the raters are all allocated to the same cluster, that is $(\eta_j, \phi_j^2, \gamma_j, \beta_j) = (\eta, \phi^2, \gamma, \beta)$ for all $j = 1, \dots, J$.

1.1 Setting

We replicate the simulation study presented in Section 6 of the paper on a small sample size with $I = 50$ subjects and $J = 10$ raters. We consider a balanced design (e.g., a fully crossed design) in which each subject is independently rated by all the raters, that is $\mathscr{R}_\rangle = 1, \dots, J$, for all $i = 1, \dots, I$. Ten independent data sets are generated from the data generative process of the *bimodal* scenario. We note that under this scenario, both models are misspecified since one cluster is assumed for raters, whereas true rater features are drawn from a bimodal distribution; see Section 6 of the paper for more details. The estimation performance of the models is compared with the same criterion used in that same Section.

1.2 Results

Table **??** gives the RMSE and the MAE of subjects' and raters' specific parameters across different datasets. The BSP model outperforms the BP and provides, on average, more accurate estimates of subjects' true scores $\{\theta_i\}_1^I$ and raters' systematic bias $\{\tau_i\}_1^J$. They recover on average the raters reliability $\{1/\sigma_i^2\}_1^J$ with the same accuracy level.

The S-RMSE and the S-MAE related to the structural parameters are reported in Table **??**. Our proposal greatly outperforms the parametric model in the estimation of raters systematic bias variance ϕ_H^2, and as a result the ICC_A. A graphical representation of the estimated true score density is given in Figure **??**. The BSP model captures the bimodal distribution of the true score and provide a better representation of the subject population heterogeneity.

		RMSE	MAE
θ	BP	2.359	0.676
	BSP	**0.727**	**0.198**
τ	BP	2.324	0.673
	BSP	**0.657**	**0.181**
$1/\sigma^2$	BP	0.012	0.003
	BSP	0.012	0.003

Table 1: Root Mean Square Error (RMSE) and Mean Absolute Error (MAE) of subjects' and raters' parameters.

.

		S-RMSE	S-MAE
μ	BP	0.199	0.186
μ_G	BSP	**0.086**	**0.065**
ω^2	BP	**6.412**	**6.218**
ω_G^2	BSP	6.424	6.240
ϕ^2	BP	8.838	7.004
ϕ_H^2	BSP	**1.328**	**1.148**
$\tilde{\sigma}$	BP	**0.118**	**0.106**
$\tilde{\sigma}_H$	BSP	0.120	0.110
ICC_A	BP	0.156	0.143
	BSP	**0.056**	**0.046**

Table 2: Standardized Root Mean Square Error (S-RMSE) and Standardized Mean Absolute Error (S-MAE) of structural parameters.

.

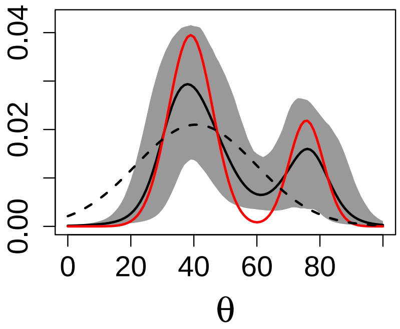

Figure 1: Subject true score density. True density (red solid line); posterior density implied by the BP model (black dotted line); pointwise posterior mean and quantile based 95% credible interval of the estimated density of BSP model.

2 Additional Results

2.1 Simulation Raters Densities

In this Section, additional plots from the simulation study of Section 6 of the paper are presented.

Figure **??** and Figure **??** show some examples of density estimates of the rater's systematic bias and reliability across different scenarios.

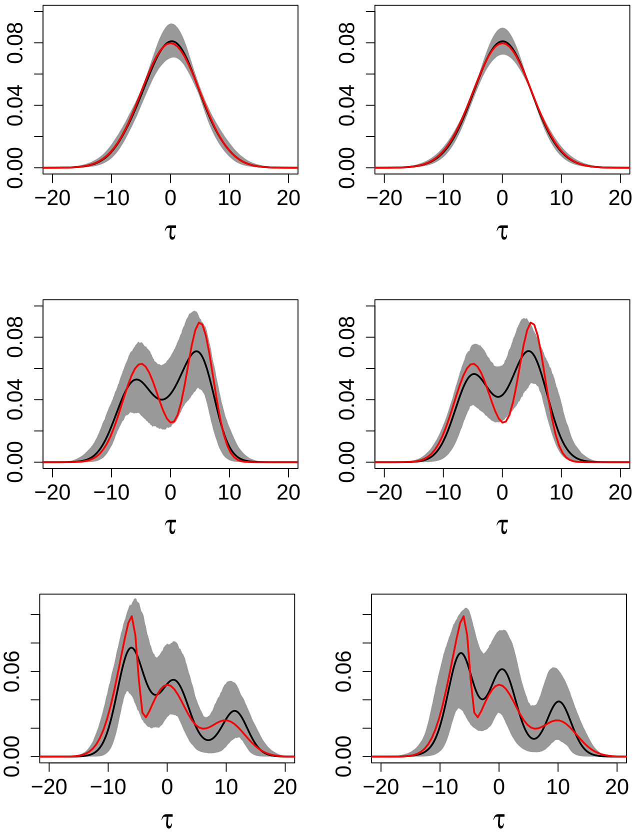

Figure 2: Illustrative examples of estimated densities of the rater's systematic bias τ under the $|\mathscr{R}_{\rangle}| = 2$ (left column) and $|\mathscr{R}_{\rangle}| = 4$ (right column) scenarios. ; the generative models are *unimodal*, *bimodal*, *multimodal*, respectively top, middle and bottom rows. The solid red lines indicate the true densities, the solid black line and the shaded grey area indicate, respectively, the point-wise mean and 95% Credible Intervals.

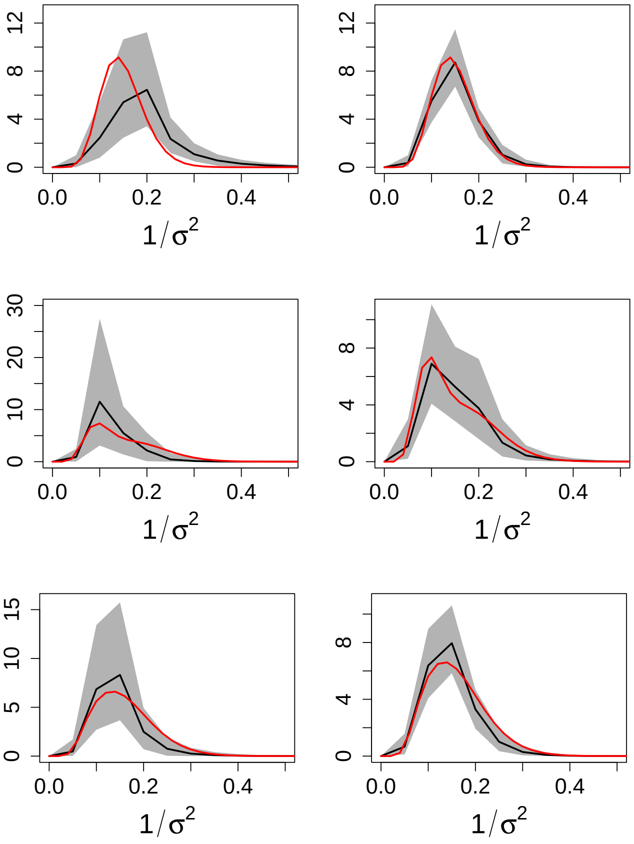

Figure 3: Illustrative examples of estimated densities of the rater's reliability ξ under the $|\mathscr{R}_{\rangle}| = 2$ (left column) and $|\mathscr{R}_{\rangle}| = 4$ (right column) scenarios; the generative models are *unimodal*, *bimodal*, *multimodal*, respectively top, middle and bottom rows. The solid red lines indicate the true densities, the solid black line and the shaded grey area indicate, respectively, the point-wise mean and 95% Credible Intervals.

2.2 Posterior Predictive Distribution

Figure **??** shows the empirical distribution and the some test statistic of replicated data from the posterior distribution of the BNP (left column) and the BP models (right column). The models were fitted to the data generated under the *multimodal* simulation scenario detailed in Section 6 of the paper. The test statistics, $T_1(y, \mu_G) = |y_{.25} - \mu_G| - |y_{.75} - \mu_G|$ and $T_2(y) = min(y)$, capture central asymmetries and tail weights, respectively.

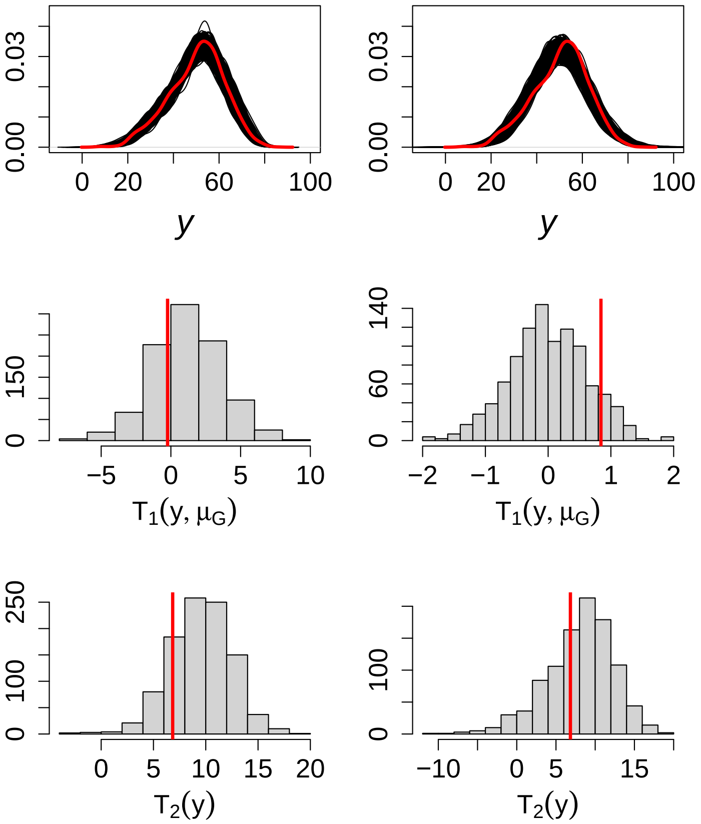

Figure 4: First row: empirical distribution of the data (red solid line) and empirical distribution of replicated data (black solid lines) from the respective BNP and BP posterior distributions (left and right columns, respectively). Second and third row: Test statistics computed on the data (red solid line) and histograms of those computed on replicated data.

2.3 Large Assessment Analysis

We report below the Base Measures' parameter estimates for the *Matura* data set analysed in Section 7 of the paper.

		Posterior median	95% Credible Interval
G_0	μ_0	29.157	$(28.122, 29.865)$
	S_0	31.432	$(27.548, 36.266)$
	w_0	13121.71	$(2215.573, 47239.08)$
	W_0	0.853	$(0.731, 0.966)$
H_0	D_0	5×10^{-4}	$(6 \times 10^{-5}, 0.039)$
	a_0	7046.009	$(931.926, 15242.96)$
	A_0	0.192	$(0.146, 0.267)$
	b_0	6748.556	$(865.8311, 15821.77)$
	B_0	13.211	$(11.625, 14.975)$
	m_0	6880.948	$(1094.299, 15784.5)$
	M_0	22.206	$(9.661, 45.528)$

Table 3: Posterior median and 95% quantile-based credible intervals of the estimated Base Measures parameters of the BNP model are reported.

.

Hold-out Validation. In addition to computing the WAIC using the same rating sample used for model estimation, as suggested by one of the reviewers, we also evaluate the predictive performance of the BP, BSP, and BNP models in a separate rating sample. In this hold-out validation procedure, the sample analyzed in Section 7 of the paper represents the *training* set, while a separate sample of the same size, randomly drawn from the remaining ratings of the same year, serves as a *test* set. We note that while the students of the two samples are different, most of the raters are the same. The log predictive density in this case has the same structure of that used in the WAIC (see Section **??**, below) but is evaluated on the new data points (i.e., the new sample of ratings); we refer to this quantity as the CV.
The CVs corresponding to the BP, BSP and BNP models are 167817.842, 67078.368 and 56099.161, respectively. This result is consistent with that presented in Section 7 of the paper.

2.4 Coarsened Ratings Model - Structural Parameters

We report in Table **??** the $S-RMSE$ and the $S-MAE$ of the structural parameter estimates across scenarios of the simulation study details in Section 8.3 of the paper. Figure **??** gives graphical representations of the subjects' and raters' individual parameter densities under the BNP and BP models.

		$\lvert\mathscr{R}_i\rvert = 2$		$\lvert\mathscr{R}_i\rvert = 4$	
		S-RMSE	S-MAE	S-RMSE	S-MAE
μ	BP	0.044	0.040	0.041	0.033
μ_G	BNP	**0.014**	**0.012**	**0.029**	**0.020**
ω^2	BP	**5.158**	**5.137**	6.371	6.359
ω_G^2	BNP	5.193	5.180	**5.794**	**5.784**
ϕ^2	BP	1.105	1.081	0.824	0.795
ϕ_H^2	BNP	**0.282**	**0.239**	**0.521**	**0.500**
$\tilde{\sigma}$	BP	**0.561**	**0.561**	**0.141**	**0.100**
$\tilde{\sigma}_H$	BNP	1.301	1.198	0.887	0.872

Table 4: Standardized Root Mean Square Error (S-RMSE) and Standardized Mean Absolute Error (S-MAE) of structural parameters of the BNP and BP model versions for coarsened ratings.
.

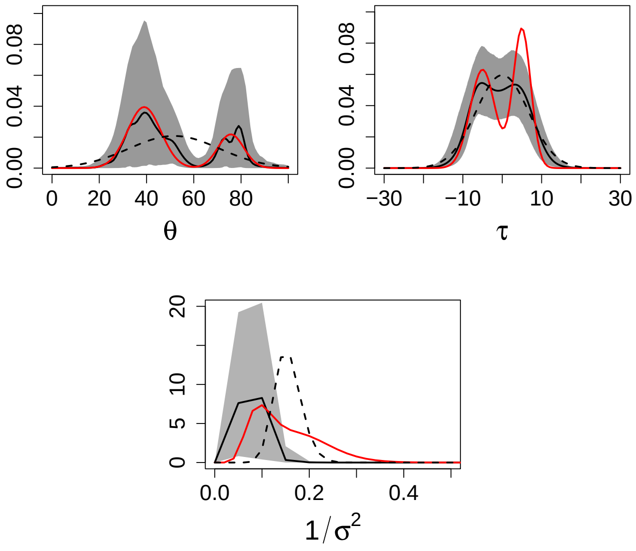

Figure 5: Subjects' and raters' specific parameter densities. True density (red solid line); posterior density implied by the BP model (black dotted line); pointwise posterior mean and quantile based 95% credible interval of the estimated density of BSP model.

3 Posterior Sampling

3.1 Gibbs sampler

1. Update true score's parameters:

   - For each subject $j = 1, \ldots, I$:

$$\theta_i|\cdot \overset{\text{ind}}{\sim} N\left(\left(\frac{1}{\omega_i^2}+\frac{|\mathscr{R}_i|}{\sum_{j\in\mathscr{R}_i}\sigma_j^2}\right)^{-1},\left(\frac{\mu_i}{\omega_i^2}+\sum_{j:\mathscr{R}_i}\frac{Y_{ij}-\tau_j}{\sigma_j^2}\right),\left(\frac{1}{\omega_i^2}+\frac{|\mathscr{R}_i|}{\sum_{j\in\mathscr{R}_i}\sigma_j^2}\right)^{-1}\right)$$

   - For each component $n = 1, ..., R$ of the mixture :

     - If $\nexists\, i : c_{1i} = n$ (if no subjects are currently assigned to the n-th component):

$$\begin{aligned}\mu_n|\cdot &\overset{\text{ind}}{\sim} N(\mu_0, S_0)\\ 1/\omega_n^2|\cdot &\overset{\text{ind}}{\sim} Ga(w_0, w_0/W_0)\end{aligned}$$

     - If $\exists\, i : c_{1i} = n$ (if at least one subject is assigned to the r-th component), the location and the scale of the n-th component:

$$\begin{aligned}\mu_n|\cdot &\overset{\text{ind}}{\sim} N\left(\left(\frac{1}{S_0}+\frac{N_{1n}}{\omega_n^2}\right)^{-1}\left(\frac{\mu_0}{S_0}+\frac{N_{1n}}{\omega_n^2}\bar{\theta}_n\right),\left(\frac{1}{S_0}+\frac{N_{1n}}{\omega_n^2}\right)^{-1}\right)\\ 1/\omega_n^2|\cdot &\overset{\text{ind}}{\sim} Ga\left(w_0+\frac{N_{1n}}{2},\ w_0/W_0+\frac{1}{2}\sum_{i:c_{1i}=n}(\theta_i-\bar{\theta}_n)^2\right)\end{aligned}$$

     where $\bar{\theta}_n = \sum\theta_i$ for $i : c_{1i} = n$ is the mean true score of the subjects assigned to component n.

   - Each subject $i = 1, ..., I$ is re-allocated into a cluster:

$$\begin{aligned}c_{1i}|\cdot &\overset{\text{ind}}{\sim} Cat(\pi_i^*)\\ \pi_i^* &= \frac{\pi_{2n}N(\theta_i|\mu_k,\omega_k^2)}{\sum_{n=1}^{R}\pi_{2n}N(\theta_i|\mu_k,\omega_k^2)}\end{aligned}$$

     where $Cat(\cdot)$ stands for Categorical distribution.

   - For each component $n = 1, ..., R-1$:

$$V_{1n}|\cdot \overset{\text{ind}}{\sim} Be\left(1+N_{1n}, \alpha_1+\sum_{l=n+1}^{R}N_{1l}\right)$$

     and $V_{1R} = 1$ for the last component.

   - The precision parameter is updated as follows:

$$\alpha_1|\cdot \sim Ga\left(R-1+a_1, b_1-\sum_{n=1}^{R-1}log(1-V_{1n})\right)$$

- The parameters of the base measure G_0 are updated:

$$\begin{aligned}
\mu_0|\cdot &\sim N\left(\left(\frac{1}{\kappa_{\mu_0}^2}+\frac{I}{S_0}\right)^{-1}\left(\frac{\lambda_{\mu_0}}{\kappa_{\mu_0}^2}+\frac{I}{R\cdot S_0}\sum_{n=1}^{R}\mu_n\right)\right)\\
S_0|\cdot &\sim IGa\left(q_{S_0}+\frac{1}{2}I,\ Q_{S_0}+\frac{1}{2}\sum_{n=1}^{R}(\mu_n-\mu_0)^2\right)\\
W_0|\cdot &\sim IGa\left(q_{W_0}+J\cdot w_0, Q_{W_0}+w_0\sum_{n=1}^{R}\frac{1}{\omega_n^2}\right)
\end{aligned}$$

The parameter w_0 is updated through the D-M algorithm introduced by **?**.

2. Update raters' systematic bias and reliability parameters:

- For each rater $r = 1,\dots,J$:

$$\begin{aligned}
\tau_j|\cdot &\overset{\text{ind}}{\sim} N\left(\left(\frac{1}{\phi_j^2}+\frac{|\mathscr{S}_j|}{\sigma_j^2}\right)^{-1}\left(\frac{\eta_j}{\phi_j^2}+\frac{\sum_{i\in\mathscr{S}_j}(Y_{ij}-\theta_i)}{\sigma_j^2}\right),\left(\frac{1}{\phi_j^2}+\frac{|\mathscr{S}_j|}{\sigma_j^2}\right)^{-1}\right)\\
1/\sigma_j^2|\cdot &\overset{\text{ind}}{\sim} G\left(1+\gamma_k+\frac{N_{2k}}{2},\frac{1+\gamma_k}{\beta_k}+\frac{1}{2}\sum_{i\in\mathscr{S}_j}(Y_{ij}-\theta_i-\tau_j)^2\right)
\end{aligned}$$

- For each component $k = 1,...,R$ of the mixture : - If $\nexists\ j : c_{2j} = k$ (if no raters are currently assigned to the k-th component):

$$\begin{aligned}
\eta_j|\cdot &\overset{\text{ind}}{\sim} N(\eta_0, D_0)\\
1/\phi_n^2|\cdot &\overset{\text{ind}}{\sim} Ga(a_0, a_0/A_0)\\
\gamma_n|\cdot &\overset{\text{ind}}{\sim} Ga(b_0, B_0)\\
1/\beta_n|\cdot &\overset{\text{ind}}{\sim} Ga(m_0, m_0/M_0)
\end{aligned}$$

  - If $\exists\ j : c_{2j} = k$ (if at least one subject is assigned to the k-th component), the location and the scale of the k-th component:

$$\eta_j|\cdot \overset{\text{ind}}{\sim} \left(\left(\frac{1}{D_0}+\frac{N_{2k}}{\phi_k^2}\right)^{-1}\left(\frac{\eta_0}{D_0}+\frac{N_{2k}}{\phi_k^2}\bar{\tau}_n\right),\left(\frac{1}{D_0}+\frac{N_{2k}}{\phi_k^2}\right)^{-1}\right) \tag{1}$$

  Algorithm 1 is detailed in Section 5.2 of the paper.

- Each rater $j = 1,...,J$ is re-allocated into a cluster:

$$\begin{aligned}
c_{2j}|\cdot &\overset{\text{ind}}{\sim} Cat(\pi_j^*)\\
\pi_j^* &= \frac{\pi_{2k}N(\tau_j|\eta_k,\phi_k^2)}{\sum_{k=1}^{R}\pi_{2k}N(\tau_j|\eta_k,\phi_k^2)}
\end{aligned}$$

- For each component $k = 1, ..., R-1$:

$$V_{2k}|\cdot \overset{\text{ind}}{\sim} Be\left(1 + N_{2k}, \alpha_2 + \sum_{l=k+1}^{R} N_{2l}\right)$$

and $V_{2R} = 1$ for the last component.

- The precision parameter is updated as follows:

$$\alpha_2|\cdot \sim Ga\left(R - 1 + a_2, b_2 - \sum_{k=1}^{R-1} log(1 - V_{2k})\right)$$

- The parameters of the base measure H_0 are updated:

$$\begin{aligned}
\eta_0|\cdot &\sim N\left(\left(\frac{1}{\kappa^2_{\eta_0}} + \frac{J}{D_0}\right)^{-1}\left(\frac{\lambda_{\eta_0}}{\kappa^2_{\eta_0}} + \frac{J}{D_0}\frac{1}{R}\sum_{k=1}^{R}\eta_k\right)\right) \\
D_0|\cdot &\sim IGa\left(q_{D_0} + \frac{1}{2}J,\ Q_{D_0} + \frac{1}{2}\sum_{k=1}^{R}(\eta_k - \eta_0)^2\right) \\
A_0|\cdot &\sim IGa\left(q_{A_0} + J \cdot a_0, Q_{A_0} + a_0 \sum_{n=1}^{R} \frac{1}{\phi_n^2}\right) \\
B_0|\cdot &\sim IGa\left(q_{B_0} + J \cdot b_0, Q_{B_0} + b_0 \sum_{n=1}^{R} \frac{1}{\gamma_n}\right) \\
M_0|\cdot &\sim IGa\left(q_{M_0} + J \cdot m_0, Q_{M_0} + m_0 \sum_{n=1}^{R} \frac{1}{\beta_n}\right)
\end{aligned}$$

The parameters a_0, b_0 and m_0 are updated through the D-M algorithm introduced by **?**.

3.2 Derivation of the D-M algorithm

We derive here the formulas for the algorithm (1) under the shape constraint (30) which are the same for all the mixture components $k = 1, \ldots, R$.
Consider the log density and the corresponding first and second derivatives at a point γ_k:

$$\begin{aligned}
log\, g(\gamma_k|U_{1k}, U_{2k}) &= U_{1k} log(U_{2k}) - log\Gamma(U_{1k}) + (U_{1k} - 1) log(\gamma_k) - U_{2k}\, \gamma_k \\
\frac{\partial}{\partial \gamma_k} log\, g(\gamma_k|U_{1k}, U_{2k}) &= \frac{U_{1k} - 1}{\gamma_k} - U_{2k} \qquad (2) \\
\frac{\partial^2}{\partial^2 \gamma_k} log\, g(\gamma_k|U_{1k}, U_{2k}) &= -\frac{U_{1k} - 1}{\gamma_k^2}. \qquad (3)
\end{aligned}$$

Given that $p(\gamma_k|\cdot) \propto p(\{1/\sigma_j^2\}_{j\in\mathscr{C}_{2k}}|\gamma_k, \beta_k, b_0, B_0)\ Ga(\gamma_k|\beta_k, b_0, B_0)$:

$$\begin{aligned}
log\ p(\gamma_k|\cdot) &= log\ p(\{1/\sigma_j^2\}_{j\in\mathscr{C}_{2k}}|\gamma_k, \beta_k, b_0, B_0)\ log\, Ga(\gamma_k|\beta_k, b_0, B_0) + const \\
&\propto N_{2k}\,(1 + \gamma_k)\, log(1 + \gamma_k) - N_{2k}\, log\Gamma(1 + \gamma_k) - (T_k + N_{2k})(1 + \gamma_k),
\end{aligned}$$

where $\{1/\sigma_j^2\}_{j\in\mathscr{C}_{2k}}$ is the sequence of reliability parameters of raters assigned to cluster k, and:

$$\begin{aligned}
\frac{\partial log\ p(\gamma_k|\cdot)}{\partial \gamma_k} &= N_{2k}\ log(1+\gamma_k)+N_{2k}-N_{2k}\ \psi(1+\gamma_k)-(T_k+N_{2k})+ \\
&\quad +\frac{b_0-1}{b_0}-B_0; \qquad (4)\\
\frac{\partial^2 log\ p(\gamma_k|\cdot)}{\partial^2 \gamma_k} &= \frac{N_{2k}}{1+\gamma_k}-N_{2k}\ \psi'(1+\gamma_k)-\frac{b_0-1}{\gamma_k^2}. \qquad (5)
\end{aligned}$$

Equating (**??**) with (**??**) and solving for U_{1k}:

$$U_{1k}=b_0+N_{2k}\ \gamma_k^2\psi'(1+\gamma_k)-\frac{N_{2k}\ \gamma_k^2}{1+\gamma_k},$$

and equating (**??**) with (**??**) and solving for U_{2k}:

$$U_{2k}=B_0+\frac{U_{1k}-b_0}{\gamma_k}-N_{2k}\ log(1+\gamma_k)+N_{2k}\ \psi(1+\gamma_k)+T_k.$$

Here $\Gamma(\cdot)$ is the Gamma function.

4 Expected Raters' reliability

Under the distributional assumption (26):

$$\sigma_j^2|\gamma,\beta \overset{\text{iid}}{\sim} IGa\left(1+\gamma,\frac{1+\gamma}{\beta}\right), \quad j=1,\ldots,J.$$

where $1+\gamma$ and $(1+\gamma)/\beta$ are respectively the shape and scale parameters of the inverse gamma. Under this parametrization, the shape parameter is always greater than one for any $\gamma>0$, which implies:

$$\mathbf{E}[\sigma_j^2|\gamma,\beta]=\frac{1+\gamma}{\beta}\frac{1}{(1+\gamma)-1}=\frac{1+\gamma}{\beta\gamma}.$$

5 WAIC formulas

In the present work, the Watanabe-Akaike Information Criteria below are used. We refer to **?** for a more detailed treatment of this index. We note that, as the parametric and the semiparametric models are nested within the BNP, the corresponding *WAICs* are reduced versions of *WAIC* used for the BNP model. More precisely, we marginalize the mixture components' parameters in estimating the out-of-sample expectation. In other words, when a mixture prior is placed over a set of parameters (e.g., the true scores), we consider replications in which the mixture indicators (i.e., the allocation of the individuals) are redrawn (**?**). This allow us to consider subjects' (and raters') heterogeneity in the model predictive performance.

BNP Model. The $WAIC$ is defined as $WAIC = -2lppd + 2p_{WAIC}$, the first term indicates the log pointwise predictive density ($lppd$); the second approximates the number of parameters in the model and serves as a complexity penalty in this formula. In hierarchical models, one has to choose which aspects of the data would vary in hypothetical replications. We want to assess the model performance in predicting the rating $y^*_{i'j'}$ given by a new rater j' to a new subject i'. This allows us to consider the structural heterogeneity of the respective populations in the predictive task, and we assume this structure to be constant across replications. This implies that we condition the $lppd$ and our prediction on the mixtures G and H of eq. (14) and (15) and consider $lppd = lppd_{G,H}$ and $p_{WAIC} = p_{WAIC_{G,H}}$ henceforth.
We define the $lppd$ as:

$$\begin{aligned} lppd &= log \prod_{i=1}^{I} \prod_{j \in \mathscr{R}_i} p(y_{ij}|G,H) \\ &= \sum_{i=1}^{I} \sum_{j \in \mathscr{R}_i} log \int_{\Lambda_1} \int_{\Lambda_2} p(y_{ij}|\lambda_1,\lambda_2) p_{\lambda_1}(\lambda_1|G) p_{\lambda_2}(\lambda_2|H) \, d\lambda_1 d\lambda_2 \end{aligned}$$

and the adjustment for the number of parameters p_{WAIC} as:

$$p_{WAIC} = \sum_{i=1}^{I} \sum_{j \in \mathscr{R}_i} Var \left[log \int_{\Lambda_1} \int_{\Lambda_2} p(y_{ij}|\lambda_1,\lambda_2) p_{\lambda_1}(\lambda_1|G) p_{\lambda_2}(\lambda_2|H) \, d\lambda_1 d\lambda_2 \right]$$

where $\lambda_1 = \theta \in \Lambda_1 = \mathbb{R}$ and $\lambda_2 = (\tau, \sigma^2) \in \Lambda_2 = \mathbb{R} \times \mathbb{R}^+$.
We compute the $WAIC$ in practice drawing S new values from $p_{\lambda_1}(\lambda_1|G)$ and $p_{\lambda_2}(\lambda_2|H)$:

$$\begin{aligned} WAIC &= -2lppd + 2p_{WAIC} &&= -2 \sum_{i=1}^{I} \sum_{j \in \mathscr{R}_i} log \left(\frac{1}{S} \sum_{s=1}^{S} p(y_{ij}|\lambda_1^s, \lambda_2^s) \right) \\ & && +2 \sum_{i=1}^{I} \sum_{j \in \mathscr{R}_i} V_{s=1}^{S} \left(log \, p(y_{ij}|\lambda_1^s, \lambda_2^s) \right) \end{aligned}$$

where $V_{s=1}^{S} a_s = \frac{1}{S-1} \sum s = 1^S (a_s - \bar{a})^2$ is the sample variance formula. For continuous ratings, the kernel of the likelihood is normal $p(y|\cdot) = N(y|\cdot)$, whereas, for coarsened (ordered) ratings it is multinomial $p(y|\cdot) = Mult(y|\cdot)$, with the probability vector given by the link function.

Semiparametric and Parametric Models. When G or H (or both in the parametric specifications) degenerate on a unique mixture component (i.e., one specifies a model with only one cluster for subjects or raters), λ_1 (and/or λ_2) is drawn from that component, and the $WAIC$ formula can be readily applied.

6 Further considerations on rater reliability under the HETOP specification

Considering the HETOP model given in (47), three different types of rating behaviors might be consistently detected for each rater $j = 1, \ldots, J$, given $K - 1$ ordered thresh-

olds:

- a consistent/ reliable behavior (as σ_j goes to zero);
- an unreliable *guessing* behavior (as σ_j goes to values such that the rating categories are equiprobable);
- an unreliable *aberrant* behavior (as σ_j goes to infinity and the grader gives “on purpose” distorted malevolent grades using extreme categories).

It might be useful to differentiate between the last two types of rating behaviors, for instance when raters are not well-trained, or to detect malevolent rating behavior.
